# Supplementary material for: Novel biomarker profiles to improve individual diagnosis and prognosis in patients with suspected inflammatory bowel disease: protocol for the Nordic inception cohort study (NORDTREAT)
Source: BMJ Open. 2024 May 15;14(5):e083144. doi: 10.1136/bmjopen-2023-083144 (PMC11097809; doi:10.1136/bmjopen-2023-083144)
Supplement: Supplementary data [file bmjopen-2023-083144supp001.pdf]

Appendix 1: Inclusion sites in the NORDTREAT Cohort study

Country	Hospital
DK	Odense University Hospital
DK	OUH Svendborg Hospital
DK	SLB Vejle Hospital
DK	SVS Esbjerg Hospital
IS	Landspitali Reykjavik
NO	Oslo University Hospital
NO	Vestfold Hospital
NO	Drammen Hospital
NO	Skien Hospital
SE	Örebro University Hospital
SE	Högland Hospital, Eksjö
