# Supplementary material for: Novel biomarker profiles to improve individual diagnosis and prognosis in patients with suspected inflammatory bowel disease: protocol for the Nordic inception cohort study (NORDTREAT)
Source: BMJ Open. 2024 May 15;14(5):e083144. doi: 10.1136/bmjopen-2023-083144 (PMC11097809; doi:10.1136/bmjopen-2023-083144)
Supplement: Supplementary data [file bmjopen-2023-083144supp002.pdf]

NORDTREAT

SOP name: General blood sampling procedure Version V1.1Approval Date: 28.03.2022

General blood sampling procedures

Concerning:

The NORDTREAT treatment strategy trial (WP2)	x
The NORDTREAT prospective cohort study (WP3)	x

Blood sampling procedure for Screening/(Baseline) and week 52
This procedure is for the following time points

Visit	Screening/ (Baseline)	12 weeks	26 weeks	52 weeks
Collected	x	x	x	x

- Blood should not be drawn when the clensing procedure for colonoscopy is ongoing or on the day of colonoscopy (due to possible effects of bowel cleansing and colonoscopy procedures)
- Use a Vacuette Butterfly 21g 19cm (green). This sterile disposable cannula with a hose facilitates sampling and prevents backflow of liquid from the PAXgene® tubes.

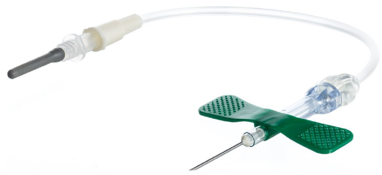

- Because of the hose, 1-2 ml of blood must be drawn in to an empty tube («throw away tube») before the first tube for biobanking is drawn.
- Then draw the blood in the following order:
  - 1. Serum tubes (for details see SOPs for Serum WP2 and Serum WP3)
  - 2. EDTA tubes (for details see SOPs for EDTA plasma WP3 and Whole blood WP3)
  - 3. PAXgene® tube (for details see SOP for PAXgene® WP3)(only at Screening/(Baseline) and week 52)

Responsible: Marte Lie Høivik

NORDTREAT

SOP name: Serum WP3Version V1.0Approval Date: 17.01.2022

Serum

Concerning:

The NORDTREAT treatment strategy trial (WP2)	
The NORDTREAT prospective cohort study (WP3)	x

This material is collected at the following time points

Visit	Screening/(Baseline)	12 weeks	26 weeks	52 weeks
Collected	x	x	x	x

Material needed

- Two Serum tubes 10 ml Vacuette (draws 9 ml blood) (455092) use containers with a red cap
- 12 tubes for biobanking (e.g. 2 ml microtubes Sarstedt 72608)

Labelling

Containers are labeled as XX-YY-ZZZ-SO-V[b] using centrally provided labels from national coordinator.

[b]XX=country code, YY=centre code, ZZZ=subject code, SO=serum observention and V=visit code (where -2=screening, 0=baseline, 12=week 12, 26=week 26, and 52=week 52))

Procedure

The serum must **be stored at -80℃ no later than two hours after the blood is drawn**.

a) Leave the tube for 60 minutes at room temperature for clot formation
b) Remove the clot by centrifugation at 2.000 x G for 10 minutes at room temperature.
c) The serum is pipetted to the following aliquots: (Of note, sufficient volume of serum should be left in the container above the pellet)
   - 12 aliquots of 500µL

   Each aliquot is labeled as XX-YY-ZZZ-SI-V[b], using centrally provided labels from the national coordinator.
d) Storage at -80℃

Responsible: Marte Lie Høivik

NORDTREAT

SOP name: Serum WP3Version V1.0Approval Date: 17.01.2022

Responsible: Marte Lie Høivik

NORDTREAT

SOP name: EDTA whole blood WP3Version V1.0Approval Date: 17.01.2022

Whole blood

Concerning:

The NORDTREAT treatment strategy trial (WP2)	
The NORDTREAT prospective cohort study (WP3)	x

This material is collected at the following time points

Visit	Baseline/inclusion	12 weeks	26 weeks	52 weeks
Collected	x			

Material needed

- One 4 ml K2EDTA Vacuette (454209 4ml)
- Two 3,5 ml Nunc tubes

Labelling

Each tube is labeled as XX-YY-ZZZ-WH-V[b], using centrally provided labels from the national coordinator

[b]XX=country code, YY=centre code, ZZZ=subject code, WH=whole blood and V=visit code where 0=baseline

Procedure sampling and handling

a) Gently mix 8-10 times immediately after sampling.
b) No centrifugation. Aliqout approximately equal volumes into two Nunc tubes.
c) The material must be stored at -80°C no later than two hours after the blood is drawn.

Responsible: Marte Lie Høivik

NORDTREAT

SOP name: EDTA plasma WP3Version V1.0Approval Date:17.01.2022

EDTA plasma

Concerning:

The NORDTREAT treatment strategy trial (WP2)	
The NORDTREAT prospective cohort study (WP3)	x

This material is collected at the following timepoints

Visit	Screening/(Baseline)	12 weeks	26 weeks	52 weeks
Collected	x	x	x	x

Material needed

- One 10 ml K2EDTA Vacuette collection tubes (455045) (violet cap)
- 9 tubes for biobanking (2 ml microtubes e.g. Sarstedt 72608)

Labelling

Each tube is labeled as XX-YY-ZZZ-P- V[b], using centrally provided labels from the national coordinator.

[b]XX =country code, YY=centre code, ZZZ=subject code, P=plasma and V=visit code (where -2= screening, 0=baseline, 12=week 12, 26=week 26, and 52=week 52)

Procedure

a) Gently mix 8-10 times immediately after sampling.
b) Centrifuge immediately at 2.000 G for 10 minutes in a room tempered centrifuge.
c) Plasma is pipetted to 9 aliquots of (at least) 500 µL, at least 1 ml of plasma should be left in the container above the pellet.
d) The material must be stored at -80°C no later than two hours after the blood is drawn.

Responsible: Marte Lie Høivik

NORDTREAT

SOP name: PAXgene® WP3Version V1.1Approval Date: 28.03.2022

PAXgene®

Concerning:

The NORDTREAT treatment strategy trial (WP2)	
The NORDTREAT prospective cohort study (WP3)	x

The material is collected at following time points:

Visit	Screening/(Baseline)	12 weeks	26 weeks	52 weeks
Collected	X			X

Material needed

- One PAXgene® (RNase free) 2.5 ml tube

Labelling

Each tube is labeled as XX-YY-ZZZ-Px-V[b], using centrally provided labels from the national coordinator.

[b]XX=country code, YY=centre code, ZZZ=subject code, Px= PAXgene and V=visit code (where -2= screening, 0=baseline and 52=week 52)

Procedure for sampling and handling of tubes

The PAXgene® (RNase free) containers are stored at room temperature (18-25°C) before use

a) PAXgene® is to be drawn as the last container (see SOP General blood sampling procedure)
b) The tube is held vertically below the patient's arm during drawing of blood
c) Use at least 10 seconds to draw the blood and to ensure that blood flow has stopped before removing the tube.
d) Gently turn 8-10 times immediately after sampling
e) Store the PAXgene® tubes upright at room temperature (18-25°C) for 2 hours
f) Freeze in an upright position at -20°C for a minimum of 24 and a maximum of 72 hours
g) Move to storage at -80°C

Responsible: Marte Lie Høivik

SOP name: Feces WP3 Version V1.0Approval Date: 17.01.2022

Feces

Concerning:

The NORDTREAT treatment strategy trial (WP2)	
The NORDTREAT prospective cohort study (WP3)	x

This material is collected at the following time points

Visit	Baseline	12 weeks	26 weeks	52 weeks
Collected	x	x	x	x

Material needed

- Stool DNA Stabilizer collection tubes Stool Collection Tubes with DNA Stabilizer
  Art nr: 1038111300 (https://www.nordicbiolabs.se/nordic-biolabs-ab/molekylarbiologi/extraction-purification/genomiskt-dna/feces-saliv/stool-dna-stabilizer-collection-tubes)
- Dry collection tube for stool samples
- Transport tubes
- Transport envelope

General information.

- Fecal samples for pathogens and Clostridium difficile are collected according to local lab routine
- At inclusion and at 52 weeks, the **fecal samples should be taken from the first stool the day before bowel cleansing is initiated (as part as the preparations for the colonoscopy). At the other visits (12 weeks, 26 weeks and extra visits) the samples should be taken on the day before or the day og the visit, if possible.**
- If the fecal sample has not obtained before colonoscopy, the patient should be instructed to provide it 3-7 days after colonoscopy.
- Detailed written instructions for sampling are included in the patient folder and should be reviewed with the patient.

Labeling

The collection tubes are labeled as XX-YY-ZZZ-FS-V[a] or XX-YY-ZZZ-FD-V[a] using centrally provided labels from the national coordinator.

[a]XX=country code, YY=centre code, ZZZ=subject code, FS=feces stabilizer and FD= feces dry and V=visit code (where 0=baseline, 12=week 12, 26=week 26, and 52=week 52)

Responsible: Marte Lie Høivik

NORDTREAT

SOP name: Feces WP3 Version V1.0Approval Date: 17.01.2022

Procedure for handing out fecal sample material

- At the screening visit, hand out the material for the baseline sample
- Provide the patient with information on how and when to collect fecal samples, **and how to record the date of collection**.
- After the Baseline/inclusion visit, hand out the remaining fecal sample kits in advance of each visit
- The fecal samples are brought by the patient to the hospital at each visit/endoscopy. If the patient is not scheduled for a visit, inform the patient about how to send the sample to the biobank (this according to national procedure)

Content in Fecal sample kit per visit

- Detailed written information for sampling
- Sampling material
- One tube with stabiliser (marked "shake well") and one dry tube.
- The collection tubes are labeled as XX-YY-ZZZ-FS-V[a] or XX-YY-ZZZ-FD-V[a] using centrally provided labels from the national coordinator

(If sendt by post: Two transport containers and one transport envelope pre-payed and pre-addressed (according to national procedure))

Sampling is described in the written information

In breaf

- In tubes with DNA preserving additives are one spoonful of feces (there is a spoon in the tube lid) is put in the tube. Lock the lid and shake well. Write the collection date on the label
- In dry tubes are The container filled half with feces. There is a spoon in the lid. Close the lid. No need for shaking. Write the collection date on the label

Put the two tubes in the transport tubes, and bring everything to the hospital visit or put everything in in the pre-addressed envelope and mail at first convenience.

Biobanking

- Record the date of fecal collection and the date of when the biobank received the sample
- The dry tubes are frozen at -80C in the collection tube
- The tubes with DNA preserving additives are frozen at -80C in the collection tube

Responsible: Marte Lie Høivik

NORDTREAT

SOP name: Feces_RNALater_WP3_Optional_Version V1.0 Approval Date: 28.03.2022

Feces

Concerning:

The NORDTREAT treatment strategy trial (WP2)	
The NORDTREAT prospective cohort study (WP3 – Danish sites)	x

The material is collected at the following time points

Visit	Baseline	12 weeks	26 weeks	52 weeks
Collected	x	x*	x*	x*

* Only IBD pt. (non-IBD will only hand in samples at baseline)

Material needed

- Stool collection tube containing RNALater solution (ThermoFisher: https://www.thermofisher.com/order/catalog/product/AM7021)
- Transport tube
- Transport envelope

General information.

- Fecal samples for pathogens and Clostridium difficile are collected according to local lab routine
- At inclusion and at 52 weeks, the **fecal sample should be taken from the first stool the day before bowel cleansing is initiated (as part of the preparations for the colonoscopy). At the other visits (12 weeks, 26 weeks and extra visits) the sample should be taken on the day before or the day of the visit, if possible.**
- If the fecal sample has not been obtained before colonoscopy, the patient should be instructed to provide it 3-7 days after colonoscopy.
- Detailed written instructions for sampling are included in the patient folder ” Prøvetagning i eget hjem – Afføringsprøve” and should be reviewed with the patient.

Labeling

The collection tubes are labeled as XX-YY-ZZZ-FR-V[a] using centrally provided labels from the national coordinator.

[a]XX=country code, YY=centre code, ZZZ=subject code, FR=feces RNA stabilizer and V=visit code (where 0=baseline, 12=week 12, 26=week 26, and 52=week 52)

Responsible: Anja Fejrskov Severino

NORDTREAT

SOP name: Feces_RNALater_WP3_Optional_Version V1.0 Approval Date: 28.03.2022

Procedure for handing out fecal sample material

- At the screening visit, hand out the material for the baseline sample
- Provide the patient with information on how and when to collect the fecal samples, **and how to record the date of collection**.
- After the Baseline/inclusion visit, hand out the remaining fecal sample kits in advance of each visit
- The fecal samples are brought by the patient to the hospital at each visit/endoscopy. If the patient is not scheduled for a visit, inform the patient how to send the sample to the biobank

Content in Fecal sample kit per visit

- Detailed written information for sampling: ” Prøvetagning i eget hjem – Afføringsprøve”
- One stool collection tube with 5 ml RNALater stabiliser
- The collection tube is labeled as XX-YY-ZZZ-FR-V[a] using centrally provided labels from the national coordinator
- Sample requisition paper with study ID and field for sample collection date.

(If sent by post: One transport container and one transport envelope pre-payed and pre-addressed)

Sampling is described in the written information

In brief

- In the tube with RNALater preserving additives four spoonful of feces from different sites are put (there is a spoon in the lid of the tube). Lock the lid and shake well. Write the collection date on the sample requisition paper in the appropriate field.
- Put the stool collection tube in the transport tube, and bring everything to the hospital visit or put everything in in the pre-addressed envelope and mail at first convenience.

Biobanking

- Record the date of fecal collection and the date of when the biobank received the sample
- The fecal sample is aliquoted in 3 micro tubes (2 ml) and frozen at -80°C

Responsible: Anja Fejrskov Severino

NORDTREAT

SOP name: Gut mucosal biopsies WP3 Version V1.1Approval Date: 28.03.2022

Gut mucosal biopsies in Allprotect®

Concerning:

The NORDTREAT prospective cohort study (WP3)	X (study biopsies)

This material is collected at the following time points

Visit	Baseline	12 weeks	26 weeks	52 weeks
Collected	x			X

Material needed

8 psc 0,5 ml containers (e.g. Sarstedt # 72.694.007) filled with 150 µl Allprotect®

Labelling

Biopsies are labeled as XX-YY-ZZZ-B(I-VIII)-V[b], using centrally provided labels from the national coordinator.

[b]XX=country code, YY=centre code, ZZZ=subject code, B=biopsy number using Roman numbers, i.e. I-VIII and V=visit code where 0=baseline and 52=week 52. For further details see table below!

Procedure for obtaining biopsies

Where to obtain biopsies

- Biopsies should be obtained from both inflamed and non-inflamed segments according to the table below
- For biopsies from inflamed mucosa, the most inflamed area in the segment should be biopsied

Handling of the biopsies

a) Standard jumbo biopsy forceps are used and one biopsy should be taken for each tube.
b) The biopsies must immediately be lowered into the liquid of Allprotect® and the biopsy must be totally covered by Allprotect® to protect DNA, RNA and proteins.
c) Incubated for a minimum of 24 and maximum of 72 hours in fridge at 2-8 °C.
d) Stored at -80 °C.

Responsible: Marte Lie Høivik

NORDTREAT

SOP name: Gut mucosal biopsies WP3 Version V1.1Approval Date: 28.03.2022

The table is used during endoscopy when taking biopsies and recording. For each segment of biopsy, make a notification in the table and upload the information into Viedoc. In UC and Crohns colitis with no non-inflamed segments of the colon, biopsies III and IV shall not be taken.

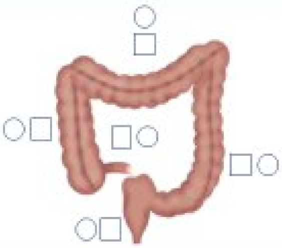

1. Tick correct diagnosis and if biopsies are taken in the table.
2. Mark site were biopsies are taken, and inflammation status on the figure.
3. Label biopsies according to "biopsy no" in the table using preprintet labels
4. Record information on site and inflammation status of biopsy 1 -8 In Viedoc.

1)N Suggested diagnose at endoscopy (tick)			2) Biopsy no			2)			2) Biopsy no			
			I	II		III	IV		V	VI	VII	VIII
	UC	Term ileum n=2			10cm prox of inflammation n=2			Rectum inflamed segment n=4				
	Crohns colitis	Term ileum n=2			10 cm prox of inflammation n=2			Most distal inflamed segment n=4				
	Ileocolonic	Term ileum n=2 (inflamed part)			10 cm prox of inflammation n=2			Most distal inflamed segment n=4				
	Ileal Crohn	Term ileum n=2 (inflamed part)			Left colon n=2			Rectum n=4				
	Normal endoscopy	Term ileum n=2			Left colon n=2			Rectum n=4				

If a biopsies are taken from this segment, mark the box with:

I= inflamed
N= non-inflamed
P= proximal

If video is recorded in this segment, tick the circle

NORDTREAT

Responsible: Marte Lie Høivik

NORDTREAT

SOP name: Snap freeze of biopsies in liquid nitrogen or FlashFreeze
Version V1.2 Approval Date: 03.11.2022

Snap freeze of biopsies in liquid nitrogen or FlashFreeze

Concerning:

The NORDTREAT treatment strategy trial	
The NORDTREAT prospective cohort study	X (study biopsies)

The material is collected at the following time points

Visit	Baseline	12 weeks	26 weeks	52 weeks
Collected	x			x

This SOP describes the processing of tissue for snap freezing with liquid nitrogen or FlashFreeze.

Material needed

Freezing in liquid nitrogen:

- Styrofoam container with dry ice
- Isolated transport container (0,5-1L) suitable for liquid nitrogen, filled approx. 2/3
- Sterile needle
- Forceps
- 6 psc. 0,5 ml micro tubes (e.g. Sarstedt # 72.694.007)
- Cryocompatible labels/marker

Additional material if freezing in FlashFreeze:

- FlashFreeze apparatus: https://www.milestonemedsrl.com/product/flashfreeze/
- Product instructions for the apparatus
- 99,9% pure ethanol

Personal Protective Equipment:

Safety glasses or face shield
Disposable latex or nitrile gloves
Gloves for handling liquid nitrogen
Clean laboratory coat

Responsible: David Füchtbauer

NORDTREAT

SOP name: Snap freeze of biopsies in liquid nitrogen or FlashFreeze

Version V1.2 Approval Date: 03.11.2022

Labelling

Biopsies are labeled as XX-YY-ZZZ-B(IX-XIIII)-V[b], using centrally provided labels from the national coordinator.

[b]XX= country code, YY= centre code, ZZZ= subject code, B= biopsy number using Roman numbers, i.e. IX-XIIII and V= visit code where 0= baseline and 52= week 52. For further details see table below!

Procedure

Liquid nitrogen:

a) Label the micro tubes with patient ID, site and visit number (Labels are pre-printed)
b) Place one biopsy in each micro tube directly after sampling - use a sterile needle to gently nudge the tissue into the tube.
c) Place without delay the micro tube in liquid nitrogen, for at least 60 sec.
d) After initial freeze, keep the cryovials on dry ice, until long-term storage at -80°C.

FlashFreeze:

If a FlashFreeze (-80°C ethanol) is used instead of liquid nitrogen, the same procedure is used, but following must be noted:

a) Set FlashFreeze apparatus to start 2 hours prior to the endoscopy. Check that the operating temperature (-76°C to -84°C) has been reached at start.
b) The vials must be in -80°C ethanol at least 120 sec. before they are moved to dry ice. (for transportation to long-term storage at -80°C)
c) Clean and defrost the apparatus as described in the product guide.

The table is used during endoscopy when taking biopsies and recording. For each segment of biopsy, make a notification in the table and upload the information into Viedoc. In UC and Crohns colitis with no non-inflamed segments of the colon, biopsies III and IV shall not be taken.

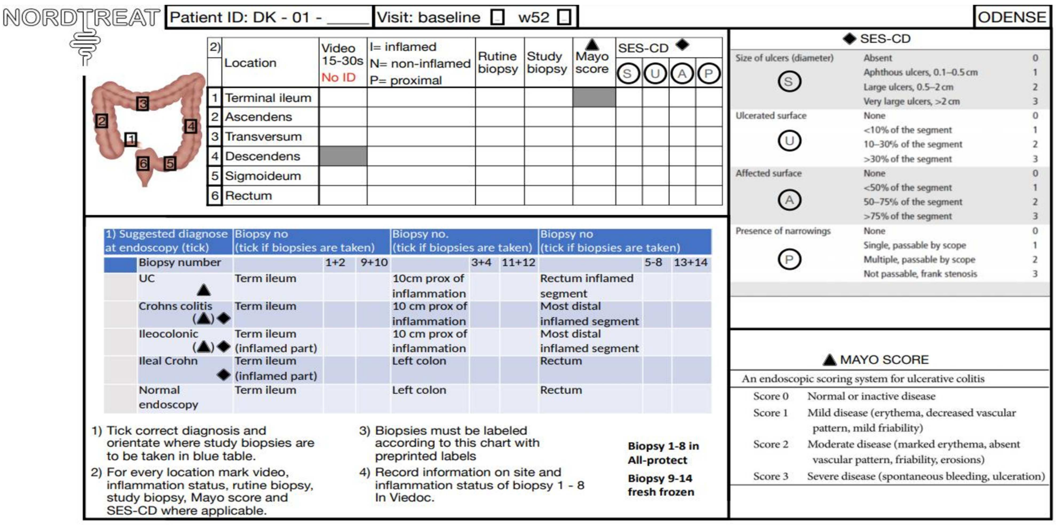

NORDTREAT Patient ID: DK - 01 - ____ Visit: baseline ☐ w52 ☐ ODENSE

2)	Location	Video 15-30s No ID	I= inflamed N= non-inflamed P= proximal	Rutine biopsy	Study biopsy	▲ Mayo score	SES-CD ◆ S	U	A	P
1	Terminal ileum									
2	Ascendens									
3	Transversum									
4	Descendens									
5	Sigmoideum									
6	Rectum									

	1) Suggested diagnose at endoscopy (tick)	Biopsy no (tick if biopsies are taken)			Biopsy no. (tick if biopsies are taken)			Biopsy no (tick if biopsies are taken)		
	Biopsy number		1+2	9+10		3+4	11+12		5-8	13+14
	UC ▲	Term ileum			10cm prox of inflammation			Rectum inflamed segment		
	Crohns colitis (▲)◆	Term ileum			10 cm prox of inflammation			Most distal inflamed segment		
	Ileocolonic (▲)◆	Term ileum (inflamed part)			10 cm prox of inflammation			Most distal inflamed segment		
	Ileal Crohn ◆	Term ileum (inflamed part)			Left colon			Rectum		
	Normal endoscopy	Term ileum			Left colon			Rectum		

1) Tick correct diagnosis and orientate where study biopsies are to be taken in blue table.
2) For every location mark video, inflammation status, rutine biopsy, study biopsy, Mayo score and SES-CD where applicable.
3) Biopsies must be labeled according to this chart with preprinted labels
4) Record information on site and inflammation status of biopsy 1 - 8 In Viedoc.

Biopsy 1-8 in All-protect

Biopsy 9-14 fresh frozen

◆ SES-CD

Size of ulcers (diameter) S	Absent	0
	Aphthous ulcers, 0.1–0.5 cm	1
	Large ulcers, 0.5–2 cm	2
	Very large ulcers, >2 cm	3
Ulcerated surface U	None	0
	<10% of the segment	1
	10–30% of the segment	2
	>30% of the segment	3
Affected surface A	None	0
	<50% of the segment	1
	50–75% of the segment	2
	>75% of the segment	3
Presence of narrowings P	None	0
	Single, passable by scope	1
	Multiple, passable by scope	2
	Not passable, frank stenosis	3

▲ MAYO SCORE

An endoscopic scoring system for ulcerative colitis

Score 0	Normal or inactive disease
Score 1	Mild disease (erythema, decreased vascular pattern, mild friability)
Score 2	Moderate disease (marked erythema, absent vascular pattern, friability, erosions)
Score 3	Severe disease (spontaneous bleeding, ulceration)

Responsible: David Füchtbauer

NORDTREAT

SOP name: Urine WP3Version V1.1Approval Date: 28.3.2022

Urine

Concerning:

The NORDTREAT treatment strategy trial (WP2)	
The NORDTREAT prospective cohort study (WP3)	x

This material is collected at the following time points

Visit	Baseline	12 weeks	26 weeks	52 weeks
Collected	x			x

Material needed

- One container suitable for collection of spot urine.
- Two containers for aliquots á 4 ml (e.g. Nunc Cryotubes 4,5ml)

Labeling

Each urine aliquot is labeled as XX-YY-ZZZ-U-V[b], using centrally provided labels from the national coordinator.

[b]XX=country code, YY=centre code, ZZZ=subject code, U=urine and V=visit code (where 0=baseline and 52=week 52)

Procedure for urine sampling and handling

a) Spot urine – taken when the blood samples are collected.
b) The urine is pipetted to 2 aliquots of 4 ml, frozen and stored at -80 °C the same day as collection
c) If aliquotation in not performed immediately, the urine should be stored in fridge locally within 2 hours after collection until aliquotation can be performed

Responsible: Marte Lie Høivik

NORDTREAT

SOP name: Hair Version V1.2 Approval Date: 28.03.2022

Hair

Concerning:

The NORDTREAT treatment strategy trial	
The NORDTREAT prospective cohort study	x

This material is collected at the following time points

Visit	Baseline	12 weeks	26 weeks	52 weeks
Collected	x			x

Material

- Scissors
- Comb
- Clamp to keep up buckle
- Clip or Hair elastic
- Aluminium foil
- Marker that can write on aluminium foil
- Tape
- Re-sealable plastic bag with ID number / name

Procedure

- Prepare a re-sealable plastic bag containing a piece of alu foil, a hair elastic or a clip
- Put hair up with the buckle
- Take a thin tuft of hair using the tip of a comb or the tip of a clamp
- Put the clip or the hair elastic around the hair ½-1 cm from the scalp (it is important to know the direction of the hair growth)
- Cut hair as close to the scalp as possible
- Keep the clip/hair elastic on and put the hair in alu foil
- Use tape to fix the clip/hair elastic to the alu foil
- Mark with a line on the alu foil, which part of the hair was closest to the scalp
- Wrap the hair in alu foil and put it in a re-sealable bag
- Remember ID number on the bag

Responsible: Anja Fejrskov Severino

SOP name: Hair Version V1.2 Approval Date: 28.03.2022

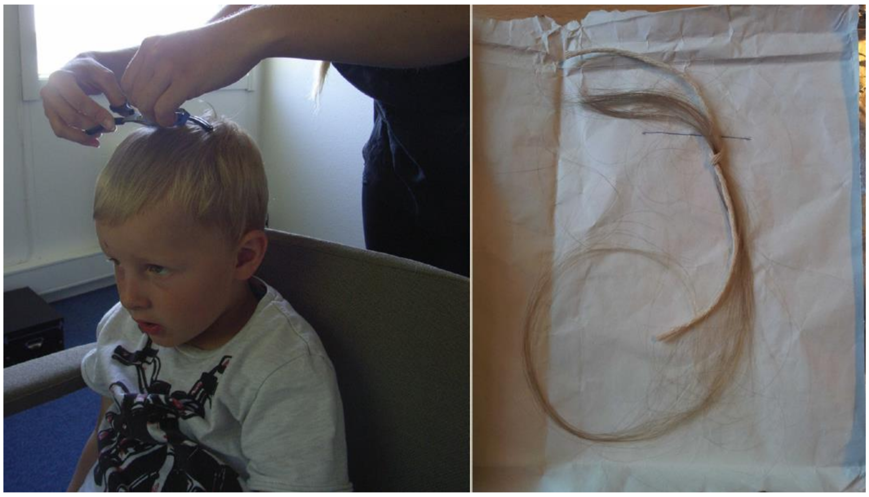

Storage of hair samples

The hair samples will be stored at room temperature, with the unique participants ID placed on the bag.

Responsible: Anja Fejrskov Severino
