# Supplementary figures and images for: Novel biomarker profiles to improve individual diagnosis and prognosis in patients with suspected inflammatory bowel disease: protocol for the Nordic inception cohort study (NORDTREAT)
Source: BMJ Open. 2024 May 15;14(5):e083144. doi: 10.1136/bmjopen-2023-083144 (PMC11097809; doi:10.1136/bmjopen-2023-083144)

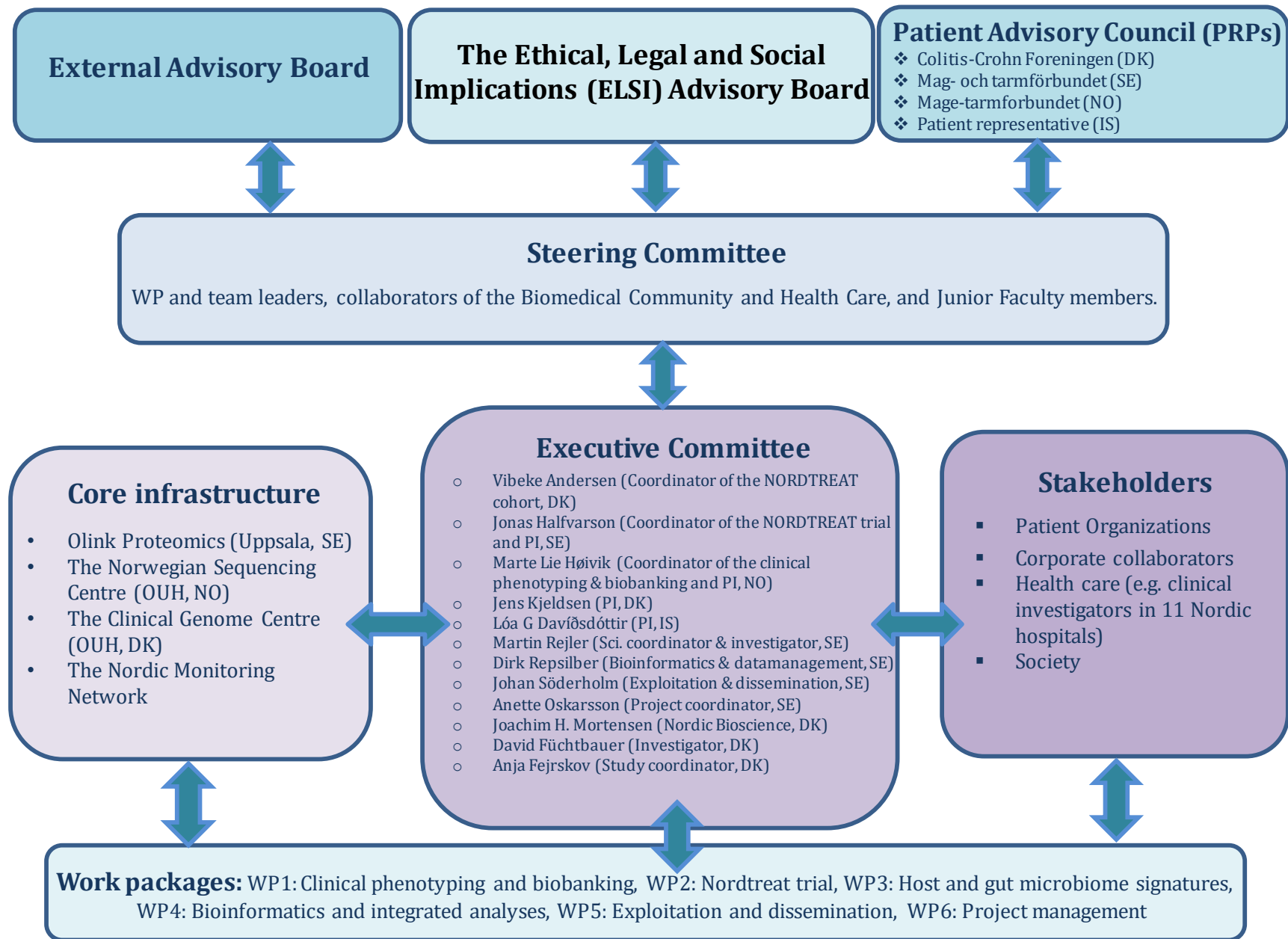

Supplement: Supplementary data [file bmjopen-2023-083144supp003.pdf]
