# Supplementary material for: Novel biomarker profiles to improve individual diagnosis and prognosis in patients with suspected inflammatory bowel disease: protocol for the Nordic inception cohort study (NORDTREAT)
Source: BMJ Open. 2024 May 15;14(5):e083144. doi: 10.1136/bmjopen-2023-083144 (PMC11097809; doi:10.1136/bmjopen-2023-083144)
Supplement: Author's manuscript [file bmjopen-2023-083144.draft_revisions.pdf]

BMJ Open

BMJ Open is committed to open peer review. As part of this commitment we make the peer review history of every article we publish publicly available.

When an article is published we post the peer reviewers' comments and the authors' responses online. We also post the versions of the paper that were used during peer review. These are the versions that the peer review comments apply to.

The versions of the paper that follow are the versions that were submitted during the peer review process. They are not the versions of record or the final published versions. They should not be cited or distributed as the published version of this manuscript.

BMJ Open is an open access journal and the full, final, typeset and author-corrected version of record of the manuscript is available on our site with no access controls, subscription charges or pay-per-view fees (http://bmjopen.bmj.com).

If you have any questions on BMJ Open's open peer review process please email info.bmjopen@bmj.com

BMJ Open

# Novel Biomarker Profiles to Improve Individual Diagnosis and Prognosis in Patients with Suspected Inflammatory Bowel Disease: Protocol for the Prospective NORDTREAT Cohort Study

| | |
|---|---|
| Journal: | *BMJ Open* |
| Manuscript ID | bmjopen-2023-083144 |
| Article Type: | Protocol |
| Date Submitted by the Author: | 12-Dec-2023 |
| Complete List of Authors: | Fejrskov, Anja; Odense University Hospital, Department of Medical Gastroenterology S; University of Southern Denmark Faculty of Health Sciences, Molecular Diagnostics and Clinical Research Unit<br>Füchtbauer, Johannes David; Odense University Hospital, Department of Medical Gastroenterology S; University of Southern Denmark, Research Unit of Medical Gastroenterology, Department of Clinical Research<br>Davíðsdóttir, Lóa ; Landspitali National University Hospital of Iceland, Department of Gastroenterology<br>Halfvarson, Jonas; Örebro University Hospital, Department of Internal Medicine<br>Høivik, Marte ; Oslo University Hospital, Department of Gastroenterology<br>Jensen, Michael ; Lillebaelt Hospital - University Hospital of Southern Denmark, Department of Internal Medicine–Gastroenterology; University of Southern Denmark Faculty of Health Sciences, Institute of Regional Health Research<br>Mortensen, Joachim ; Nordic Bioscience A/S, Gastrointestinal Diseases<br>Nielsen, Lene; Hospital South West Jutland, Research Unit of Medical gastroenterology and hepatology<br>Rejler, Martin; Jönköping University, Jönköping Academy for Improvement in Health and Welfare; Futurum Academy of Health and Care,  Futurum-Academy for Healthcare<br>Repsilber, Dirk; Örebro University, School of Health and Medical Sciences<br>Söderholm, Johan; Linköping University, Department of Biomedical and Clinical Sciences<br>Aalykke, Claus; Odense University Hospital, Internal Medicine and Emergency Department<br>Andersen, Vibeke; University of Southern Denmark Faculty of Health Sciences, Molecular Diagnostics and Clinical Research Unit; University of Southern Denmark, Institute of Molecular Medicine<br>Christensen, Robin; Parker Instituttet, Section for Biostatistics and Evidence-Based Research; University of Southern Denmark, Research Unit of Rheumatology, Department of Clinical Research<br>Kjeldsen, Jens; Odense University Hospital, Department of Medical Gastroenterology S; University of Southern Denmark, Research Unit of Medical Gastroenterology, Department of Clinical Research |
| Keywords: | Inflammatory bowel disease < GASTROENTEROLOGY, Prognosis, Gastroenterology < INTERNAL MEDICINE, Endoscopy < |

| | |
|---|---|
| | GASTROENTEROLOGY, Adult gastroenterology < GASTROENTEROLOGY |
| | |

SCHOLARONE™
Manuscripts

# Novel Biomarker Profiles to Improve Individual Diagnosis and Prognosis in Patients with Suspected Inflammatory Bowel Disease: Protocol for the Prospective NORDTREAT Cohort Study

**Authors:** Fejrskov A [1, 2, 3, *], Füchtbauer JD [1, 4, 5, *], Davíðsdóttir LG [6], Halfvarson J [7], Høivik ML [8], Jensen MD [9, 16], Mortensen JH [10], Nielsen LN [11], Rejler M [12, 13], Repsilber D [14], Söderholm JD [15], Aalykke C [4], Andersen V [2, 16, 17, 18, °], Christensen R [3, 19, °], Kjeldsen J [1, 5, °]

* Share first authorship ° Share last authorship

For numbered affiliations and open researcher and contributor ID (ORCID), see end of article.

**Correspondence to:** PhD fellow Anja Fejrskov; Anja.Fejrskov@rsyd.dk; PhD fellow Johannes David Füchtbauer; David.Fuchtbauer@rsyd.dk

**Key words:** IBD, inflammatory bowel disease, ulcerative colitis, Crohn's disease, biomarkers, diagnosis, prognosis, personalised medicine.



For peer review only - http://bmjopen.bmj.com/site/about/guidelines.xhtml

ABSTRACT

**Introduction.** Inflammatory bowel disease (IBD), including ulcerative colitis and Crohn´s disease, can be challenging to diagnose, and treatment outcomes are difficult to predict. In the NORDTREAT cohort study, a Nordic prospective multicentre study, we aim to identify novel molecular biomarkers of diagnostic value by assessing the diagnostic test accuracy (cross-sectionally), as well as the prognostic utility when used as prognostic markers in the long-term (cohort study). In the diagnostic test accuracy study, the primary outcome is a successful diagnosis using one or more novel index tests at baseline compared to the ECCO criteria as the reference standard. The composite outcome of the prognostic utility study is "severe IBD" within 52 weeks from inclusion, defined as one or more of the following three events: IBD-related surgery, IBD-related hospitalisation or IBD-related death.

**Methods and analysis.** Patients referred on suspicion of IBD will be recruited to this longitudinal observational study, a collaboration between eleven inclusion sites in Denmark, Iceland, Norway, and Sweden. Inclusion will occur from February 2022 until December 2023 with screening and baseline visits for all participants and three outcome visits at weeks 12, 26 and 52 after baseline for IBD-diagnosed patients. Biological material (blood, faeces, biopsies, urine, and hair), clinical data, and lifestyle information will be collected during these scheduled visits.

**Ethics and dissemination** We aim at exploring novel biomarkers to improve diagnostic accuracy and prediction of disease progression, thereby improving medical therapy and the quality of life for patients with IBD.

The study is approved by the Ethics Committee (DK: S-20200051, v1.4, 16.10.2021; IS: VSNb2021070006/03.01, NO: 193064; SE: DNR 2021-05090) and the Danish Data Protecting Agency (20/54594). Results will be disseminated through peer-reviewed journals, patient associations and presentations at international conferences.

Clinical trial registration number NCT05414578.




For peer review only - http://bmjopen.bmj.com/site/about/guidelines.xhtml

## Strengths and limitations of this study

- All participants included on suspicion of IBD and treatment naïve
- Strong international collaboration and extensive inclusion area with 11 sites in four Nordic countries
- Prospective, longitudinal study design with rigorously collected biological material and data on disease activity and lifestyle

- Patient heterogeneity (four countries)
- Ascertainment bias; as some severe patients may not be eligible for inclusion if acute treatment is initiated

## INTRODUCTION

Inflammatory bowel disease (IBD), primarily ulcerative colitis (UC) and Crohn´s disease (CD) are idiopathic disorders characterised by chronic gastrointestinal inflammation and relapsing symptoms like diarrhoea, abdominal pain, and fatigue. A large proportion of the patients experience severe disease manifestations like bowel obstruction, fistulising disease, liver disease, and colorectal cancer. Additionally, extra-intestinal manifestations occur in 15-40%.[1] Consequently, hospitalisations, surgical interventions, recurring sick leave and life-long medication severely impact the lives of patients and families. Even though the incidence of IBD appears to have reached a plateau in most Western European countries, it is rapidly increasing in many newly industrialised countries.[2] The prevalence of IBD has increased substantially in the last three decades, exceeding 0.5 % in many European countries.[2–4] An estimated 1.3 million people in Europe suffer from IBD, imposing a substantial health, social, and economic burden.[1] There is a need to improve the management of IBD.

Diagnosis of IBD can be cumbersome as a single reference standard does not exist.[5–7] Furthermore, IBD is a heterogeneous disease and its course differs substantially between



For peer review only - http://bmjopen.bmj.com/site/about/guidelines.xhtml

patients. The lack of vigorous biomarkers for individual disease characterisation potentially leads to delayed diagnosis, organ damage, worsened outcome, increased mortality, and an ever-increasing disease burden.[8] Personalised medicine (PM) could be a suitable strategy for optimising both diagnostics and treatment of IBD.[9] The vision of the International Consortium for Personalised Medicine (ICPerMed) describes "*an approach to medicine that integrates an individual's characteristics for early disease diagnosis, prognosis, optimal choice of treatment, accurate disease risk estimation, and targeted prevention*".[10] In gastrointestinal diseases, PM is based upon advancements in biomarker detection in a variety of omics-platforms, including genomics, transcriptomics, microbiomics, proteomics, and metabolomics, as well as body imaging methods, e.g., computed tomography (CT) scans, and magnetic resonance imaging (MRI) scans. Recent advances has led to the identification of several potential biomarkers for diagnosis and treatment response in IBD.[11–17] However, they are not in use in clinical practice. Even though early diagnosis and efficient treatment strategies for IBD are cornerstones for reducing irreversible organ damage, identifying biomarkers that can efficiently support clinical decision-making is still warranted.

This protocol article introduces a prospective clinical study that aims to identify biomarkers associated with the diagnostic accuracy of IBD and the utility of novel prognostic biomarkers in clinical outcomes with various disease manifestations. Notably, the study evaluates biomarkers in a relevant clinical setting, i.e. among patients referred to the hospital on suspicion of IBD and diagnosed according to the ECCO Criteria.[5]

## Aims

This study aims, on the one hand, to improve accuracy in diagnosing IBD in the form of a *diagnostic test accuracy study*. The main hypothesis is that biochemical markers can differentiate individuals referred to a gastroenterology department with suspected IBD into individuals with (IBD) and without (non-IBD) diagnosis. The primary aim is to identify molecular biomarkers (e.g., from blood and stools) for discriminating between individuals diagnosed with IBD (CD, UC, and unclassified inflammatory bowel disease [IBD-U]) and non-IBD. Secondary aims include investigating whether clinical, genetic, microbiome and lifestyle



For peer review only

information and combinations thereof (e.g., gene-environment interaction analyses) are associated with IBD diagnosis.

On the other hand, the main hypothesis of the prognostic *utility study* is that prognostic factors can distinguish different pathways of disease progression. The primary aim of this study is to identify prognostic biomarkers that can define different IBD disease courses. The secondary aims are to evaluate whether including information on clinical and lifestyle factors and combinations thereof can improve prognostic value.

For peer review only

## METHODS and ANALYSES

### Design

The NORDTREAT cohort study is a Nordic multicentre collaboration on diagnostic factors and prognostic factors of IBD among patients referred to the hospital with suspected IBD. All referred patients will be carefully evaluated to ensure that patients with suspected IBD are invited to participate in the study at their first clinic visit. Informed consent will be obtained at the screening visit (week -4). After study material collection (i.e. blood, stool, biopsies, hair, urine, questionnaires, and patient-reported outcome measures [PROMs]) and diagnostic evaluation, IBD diagnosis will be established or rejected at the baseline visit (week 0). IBD patients will be followed until week 52 with three additional visits (week 12, 26, and 52), whereas non-IBD patients will complete questionnaires at baseline visit and week 52 (**Figure 1**).

Figure 1: Schedule for patients with a suspected IBD diagnosis



For peer review only - http://bmjopen.bmj.com/site/about/guidelines.xhtml

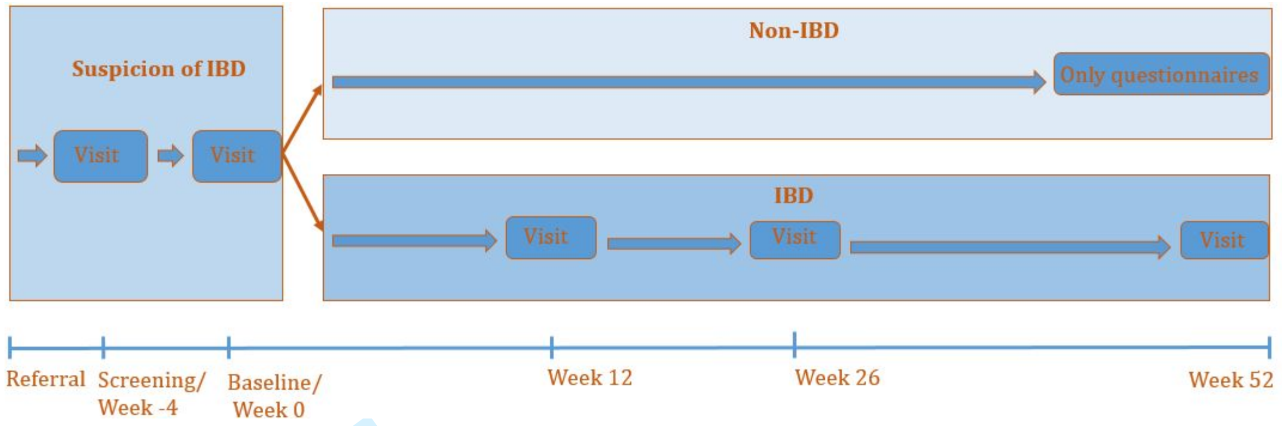


The prospective study consists of a cross-sectional part with focus on diagnosis and a longitudinal cohort study (52 weeks) with focus on prognosis (i.e., a prognostic utility study). The inclusion of patients into the NORDTREAT cohort study will be reported as a flowchart (**Figure 2**).

The objective of the *diagnostic test accuracy study* is to determine the sensitivity and specificity of biomarkers of interest (baseline measurements) to diagnose IBD when applying the ECCO Diagnostic criteria as the reference standard.[5]

The objective of the *prognostic utility study* is to examine whether the biomarkers of interest predict severe disease outcomes in patients with IBD. The proportion of patients who encounter severe IBD is defined as those experiencing IBD-related surgery, IBD-related hospitalisation (except planned procedures), or IBD-related death within 52 weeks of inclusion.

Figure 2: Flowchart for patients assessed eligible for inclusion in the NORDTREAT cohort study



For peer review only - http://bmjopen.bmj.com/site/about/guidelines.xhtml

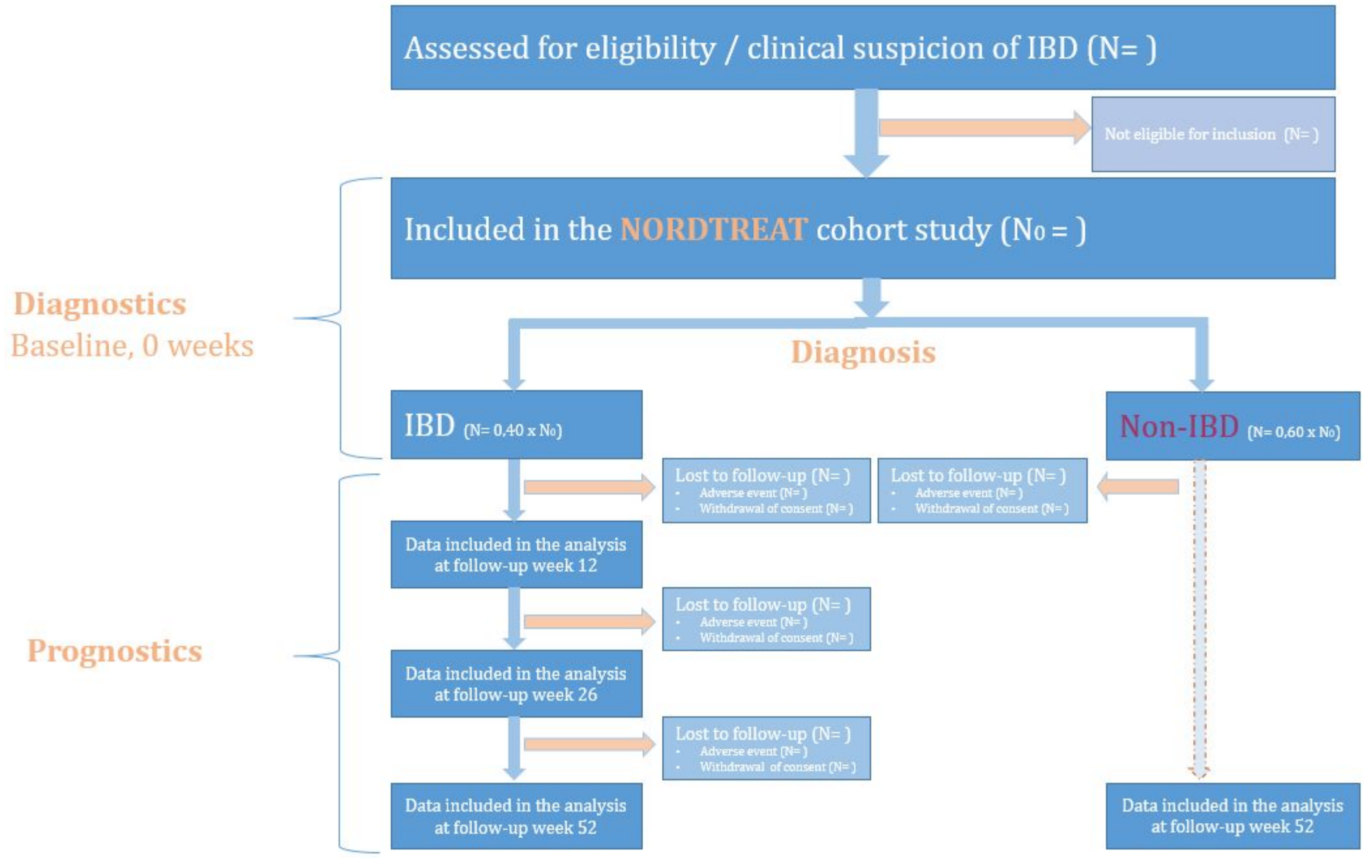


Setting

This Nordic multicentre study is a collaboration between gastroenterology departments in 11 Nordic hospitals (**Appendix 1**). The study enrolment will occur between 7 February 2022 and 31 December 2023. The first patient's first visit (FPFV) was conducted in Oslo University Hospital in February 2022, and the first patient's last visit (FPLV) was in February 2023. All inclusion sites will conduct their last patient's first visit (LPFV) and last patient's last visit (LPLV) in December 2023 and december 2024, respectively.

Patient characteristics and eligibility criteria



For peer review only - http://bmjopen.bmj.com/site/about/guidelines.xhtml

Inclusion criteria: Patients ≥18 years of age referred to a gastroenterology department with suspected IBD who give written informed consent to participate in the study. Exclusion criteria: Patients with a previous diagnosis of CD, UC or IBD-U or patients unable to provide informed consent or comply with protocol requirements, e.g. due to recreational drug or alcohol misuse.

The clinical data and lifestyle information collected for the study will be presented in tabular form (see **Table 2**) and include:

**Clinical interview.** Demographics (sex, age, marital status, educational level, occupational status, and smoking), disease history (previous disease (including debut of gastrointestinal symptoms) and surgery (abdominal and perianal), occurrence of intestinal complications (defined as strictures and fistulas), occurrence of perianal disease, extra-intestinal manifestations, medication (past and present), family history (of IBD and colorectal cancer) and at the week 52 visit: relapses and surgery (abdominal and perianal) are recorded.

**Clinical examination** includes a perianal examination of study subjects with perianal CD (accounting for the number of draining and non-draining perianal fistula).

**Vital signs** include measurement of height, weight, heart rate, blood pressure, and temperature.

**Endoscopy.** Standardised scoring systems will record macroscopic findings from the ileocolonoscopy: For patients with UC, the Endoscopic Mayo Score per segment is applied, i.e. ascending-, transverse-, descending-, sigmoid colon and rectum.[18] For patients with CD, the Simple Endoscopic Score for Crohn's Disease (SES-CD) per segment is applied, i.e. terminal ileum, ascending-, transverse-, left colon and rectum.[19] Patients diagnosed with IBD-U at baseline will be assessed with the Endoscopic Mayo Score per segment and the SES-CD per segment. The endoscopy is recorded on video (approximately 20 seconds per segment). Furthermore, standard routine biopsies from the ileum, ascending-, transverse-, descending colon, and rectum will be obtained.

**Medical records.** Routine clinical information, clinical findings and procedures (endoscopic and surgical), test results (i.e. histology, radiology [including small bowel MRI, capsule



For peer review only - http://bmjopen.bmj.com/site/about/guidelines.xhtml

endoscopy, CT scan, and ultrasonography], microbiology, and biochemistry), diagnosis, treatment and complications including hospitalisations will be retrieved from the medical records.

**Montreal classification.** Based on results from the routine examinations, the study subjects will be classified according to the internationally accepted IBD classification system, the Montreal Classification.[20]

**Lifestyle information, Disease activity index, Disease questionnaires and PROMs.** Participants will complete validated questionnaires on disease activity, quality of life, including **EQ-5D-5L**[21] (measure of e.g. mobility, personal care, usual activities, pain/discomfort, and anxiety/depression), **SF-36**[22] (including perception of health, limitations in daily and social activities, general mental health (psychological distress and well-being), and vitality (energy and fatigue) within the last 4 weeks), **IBDQ**[23,24] (disease impact on patients daily functioning and quality of life (e.g. abdominal pain, sleep, incontinence, ability to work, and conduct social activities within the last 2 weeks), **IBD-F**[25] (disease specific questionnaire comprising five questions about the severity of fatigue (Part 1) and **ROME IV**[26] (The Rome IV Diagnostic Questionnaire for Functional Gastrointestinal Disorders in Adults). The electronic case report form (eCRF) platform Viedoc™ (Viedoc Technologies, Uppsala Sweden) is applied, and in Denmark REDCap (Vanderbilt University, Nashville, TN, USA) is used for diet questionnaires.

Before each visit, patients with IBD will register clinical disease activity, Harvey-Bradshaw Index (**HBI**)[27,28] (diary for patients with CD including two clinical parameters: abdominal pain and number of liquid or soft stools), or **Partial Mayo Score** (diary for patients with UC or IBD-U including the two non-invasive components of the full Mayo Score [stool frequency and rectal bleeding). All participants will perform diet questionnaires at baseline and week 52.[29]

Table 2. Overview of clinical and lifestyle information collected according to visits (weeks after diagnosis)



For peer review only - http://bmjopen.bmj.com/site/about/guidelines.xhtml

| | IBD | | | | non-IBD | |
|---|---|---|---|---|---|---|
| Week | - 4 / 0 | 12 | 26 | 52 | - 4 /0 | 52 |
| **Clinical information** | | | | | | |
| Clinical interview and examination | x | x | | x | x | |
| Vital signs | x | x | x | x | x | |
| Endoscopy | x | | | x | x | |
| Medical records | x | x | | x | x | |
| Montreal classification | x | | | x | | |
| **Lifestyle information, Disease activity index and PROMs** | | | | | | |
| SF36 | x | x | x | x | | |
| IBDQ | x | x | x | x | | |
| EQ-5D-5L | x | x | x | x | | |
| IBD-F | x | x | x | x | x | x |
| HBI (CD only) | x | x | x | x | | |
| Partial Mayo score (UC only) | x | x | x | x | | |
| ROME IV | x | x | x | x | x | x |
| Diet | x | | | x | x | x |

Abbreviations: SF36: Short Form 36, IBDQ: Inflammatory Bowel Disease Questionnaire, IBD-F: Inflammatory Bowel Disease Fatigue (Part 1) and HBI: Harvey-Bradshaw Index.

**Biological material** (blood, biopsies, faeces, hair and urine) will be collected at the scheduled visits (**Table 3**):

At baseline, all study subjects have blood samples (whole blood [4ml], plasma [10ml], serum [20ml] and Paxgene blood RNA system [2,5ml]) and biopsies collected (8 biopsies from predefined locations collected in Allprotect reagent and 6 biopsies from predefined locations snap frozen in liquid nitrogen or FlashFREEZE [Milestone Medical, product no. 100780 from Axlab A/S, DK], ensuring a standardised freezing procedure at -80°C). Furthermore, the fecal



For peer review only - http://bmjopen.bmj.com/site/about/guidelines.xhtml

samples collected at the patient's home will consist of: one stool collection tube with RNALater, DNA stabiliser, and a dry sample, respectively, as well as a tuft of hair and urine (8ml).

Blood samples will not be collected on the day of the ileocolonoscopy, and fecal samples will not be collected during the colonic cleansing to minimise any potential influence from the standard cleansing procedure conducted before an endoscopy.

On follow-up visits at week 12 and week 26 all patients diagnosed with IBD will have blood samples (plasma [10ml] and serum [20ml]) and fecal samples (one stool collection tube with RNALater, DNA stabiliser and a dry sample, respectively) collected. At the week 52 visit for IBD patients, biological material similar to baseline will be collected (except for whole blood). The biological material will be stored at local facilities according to national procedures. In Denmark long-term storage (biobanking) is at Odense Patient data Explorative Network (OPEN).

Table 3: Overview of the biological material collected in the study

| Biological material | Specifications | Baseline IBD and non-IBD | Week 12 IBD | Week 26 IBD | Week 52 IBD |
|---|---|---|---|---|---|
| Blood | Whole blood (EDTA-K2) | x | | | |
| | Plasma (EDTA-K2 ) | x | x | x | x |
| | Serum | x | x | x | x |
| | Paxgene | x | | | x |
| Biopsies | Allprotect reagent | x | | | x |
| | Snap frozen | x | | | x |
| Fecal samples | DNA stabiliser | x | x | x | x |
| | Dry | x | x | x | x |
| | RNALater | x | x | x | x |
| Hair | 10-20mg | x | | | x |
| Urine | Spot urine | x | | | x |



For peer review only - http://bmjopen.bmj.com/site/about/guidelines.xhtml

Primary endpoint(s)

The primary endpoint for the cross-sectional *diagnostic test accuracy study* is diagnosis according to the reference standard from ECCO criteria,[30] i.e. IBD (including CD, UC, IBD-U) or non-IBD (patients not diagnosed with IBD) at referral.

The primary endpoint for the longitudinal *prognostic utility study* is a composite outcome measure. We will include multiple (three mutually associated) endpoint components combined as a Boolean function; this will be our primary outcome measure derived from randomised trials while also anticipating that the approach will be associated with increased statistical efficiency (e.g. power). The composite outcome broadly consists of the proportion of patients who experience severe IBD over 52 weeks after inclusion, defined as IBD-related surgery, IBD-related hospitalisation (except planned procedures) or IBD-related death.

Key secondary endpoints in the Prognostic Utility Study

Major secondary outcomes assessed and analysed 52 weeks from baseline will include IBD-related surgery (number [no.] in %), IBD-related hospitalisation (no., %), IBD-related deaths (no., %) as well as non corticosteroid-free (local acting systemic administered [budesonide] and systemic steroids [prednisolone]) clinical remission at week 12.[31] Clinical remission in CD is defined as patient-reported outcome 2 (abdominal pain ≤ 1 and stool frequency ≤ 3) or Harvey Bradshaw Index < 5. Clinical remission in UC is defined as patient-reported outcome 2 (rectal bleeding = 0 and stool frequency = 0) or Partial Mayo score (< 3 and no score >1). Non corticosteroid-free endoscopic remission is assessed at week 52[31] where endoscopic remission in CD is defined as Simple endoscopic score < 3 points or absence of ulcerations (e.g. SES-CD ulceration subscore = 0). Endoscopic remission in UC is defined as Mayo endoscopic score = 0 points or UC endoscopic index of severity ≤ 1 point. Disease impact on patient's life will be assessed by the 32-item version of Inflammatory Bowel Disease Questionnaire (IBDQ) score (number (%) ≤ 170) and the numerical change of the SF36 subscales.



For peer review only - http://bmjopen.bmj.com/site/about/guidelines.xhtml

Other secondary endpoints in the Prognostic Utility Study

The composite secondary outcome is the proportion of patients who experience IBD complications within 52 weeks after inclusion, defined as the cumulative number of patients who experience one of the following (*i*) need for medication (immunomodulators, biologics or steroids) between diagnosis and one year, (*ii*) need for surgery between diagnosis and one year, (*iii*) need for hospitalisation between diagnosis and one year or (*iiii*) relapse between diagnosis and one year. Furthermore, the cumulative number of flares, adverse drug reactions, numerical change in the Fatigue Questionnaire, and cumulative glucocorticoid exposure will be assessed. Fecal calprotectin > 250 µg/g (no., %), time to start of biological therapy, time to IBD-related surgery or hospitalisation, and CD-related surgery (stenosis) will also be measured.

Mid-term and long-term complications in the Prognostic Utility Study

IBD-related hospitalisations will be assessed and defined as a composite endpoint combining the total number of IBD-related hospitalisations (including re-admissions) and the cumulative length of hospital stay. Bowel damage in patients with CD (Lémann index for patients with an MRI), UC-related surgery (colectomy), CD-related surgery (no., %), IBD-related hospitalisations (no., %), and change in disease extent in UC (with a focus on macroscopic proximal disease extent) will be assessed among the mid-term complications. Among the long-term complications, the following three endpoints will be measured: gastrointestinal and extra-intestinal dysplasia (no., %), cancer (gastrointestinal and others in no., %), and mortality (no., %).

Statistical methods

Referring to earlier studies, approximately 40% of a population referred with suspected IBD will have the diagnosis confirmed according to the ECCO criteria (or comparable).[5,32] Cost and practical issues will restrict the sample size to approximately 800 patients, with an expected 80% participation rate.



For peer review only - http://bmjopen.bmj.com/site/about/guidelines.xhtml

In the *diagnostic test accuracy study*, we will compare two independent binomial proportions, using the likelihood ratio statistic with a Chi-square approximation and two-sided significance level (0.05). With an expected sample size of 640 participants (assuming a biomarker positivity-to-negativity ratio of 1 to 1) we will reach an approximate statistical power of 100%, assuming the proportion of patients diagnosed with IBD is 80% and 20% in each biomarker stratum, respectively. If we include 640 study participants, we will potentially ("rule of thumb") be able to build a multivariable statistical model with as many as 64 covariates.. Thus, we will examine a variety of biochemical variables for diagnosing IBD. The expectation is that a proportion of 100-200 preliminary biomarkers will be evaluated in this model to distinguish non-diagnostic markers and preliminary biomarkers that can be replicated. When applying a significance level of 0.05, each biomarker has a 5% risk of being replicated by chance. Thus, in the evaluation of, e.g. 100-200 biomarkers five to ten biomarkers may be replicated by chance. In contrast, the risk of not replicating "true" biomarkers with low effects is estimated to be approximately 20%. All replicated biomarkers will be built into a prediction model that will be optimised and evaluated in the replicative part of the cohort.. The primary hypothesis is that the biomarker signature will be able to characterise the participant population into two phenotype groups.

In the *prognostic utility study*, we expect a sample size of 256 IBD patients and a 10% event rate for severe IBD within 52 weeks.[33,34] If we assume a 15% event rate for the biomarker positive group and a 5% event rate for the biomarker negative group, and anticipate an even 1:1 biomarker positive-to-negative distribution, approximately 80 patients in each group will increase the statistical power above the 90% threshold.

**Comparison of measures of diagnostic accuracy:** The basic measures of quantifying the diagnostic accuracy of a test will be the diagnostic Odds Ratio, sensitivity, and specificity measures. From these measures, we will calculate the likelihood ratio (LR), the ratio of the probability of the index test result among patients who have IBD to the probability of the same test among patients who do not have IBD (non-IBD). Since all diagnostic tests will be performed



For peer review only

For peer review only - http://bmjopen.bmj.com/site/about/guidelines.xhtml

on each patient, paired data results and methods that account for the correlated binary outcomes are necessary (e.g., McNemar's test).

**Prognostic factor research (the prognostic utility study):** The primary statistical analysis will be based on the proportion of participants experiencing severe IBD among biomarker-positive versus biomarker-negative patients. The efficacy of the prognostic biomarker(s) will be considered as the proportion of the patients with different disease outcomes among the biomarker-positive patients compared to the biomarker-negative patients. The associations of the suspected important molecular biomarkers with other variables will be tested with nonparametric tests: with Spearman's rank correlation (rs) for continuous variables and the Wilcoxon rank-sum test or Kruskal-Wallis test, including a Wilcoxon-type test for trend across ordered groups where appropriate, for categorical variables.

Logistic regression models will be utilized with individual markers as the exposure variables and composite clinical severity as the outcome (dependent variable). The analyses will be adjusted simultaneously for sex, age, and prescribed therapy. Potential interactions between biomarker status (positive/negative) and specific drug type will be evaluated too. Covariates comprise of various measures within genetics, transcriptomics, microbiomes, and proteomics. We will attempt to develop prediction models containing several conventional risk factors (i.e., age, sex, type of drug therapy) with or without any novel biomarkers and calculate improvements in predictive ability using risk discrimination and reclassification measures. We will use a 2-stage approach that allows for the examination of centre heterogeneity through the calculation of the C-index, a measure of risk discrimination, and changes within each centre before pooling results.

We will attempt to develop multivariable prediction models relating multiple predictors for a particular individual to the probability of or risk for the presence (diagnosis of IBD at baseline) or future occurrence (prognosis) of a particular outcome, such as intestinal surgery within the first year. Predictors such as biomarkers are covariates, explored as prognostic factors —more statistically—these are independent variables.



For peer review only - http://bmjopen.bmj.com/site/about/guidelines.xhtml

When reporting the *diagnostic test accuracy study*, we will adhere to the STARD guideline[35] and the TRIPOD statement,[36] and when reporting the *prognostic utility study* we will apply the STROBE[37] as well as the TRIPOD statement.[36] Before any analyses are initiated an elaborated statistical analysis plan (SAP) indicating explicit figures and tables will be provided.

Project organisation

The NORDTREAT partnership builds on extensive professional experience, a strong track record of collaborative clinical projects and participation in a number of European and global consortia (e.g., the IBD Character Consortium, the SYSCID Consortium and the International IBD Genetic Consortium).

The NORDTREAT cohort study is conducted in parallel and as part of the NORDTREAT trial (a biomarker-stratified randomised controlled trial [RCT]). The aim of the NORDTREAT RCT is to demonstrate that personalised therapy can be applied to patients with IBD, by assessing if a top-down treatment can improve clinical outcomes in IBD patients with an increased risk of poor disease course (https://clinicaltrials.gov/ct2/show/NCT05180175).

The operational organization of the NORDTREAT study is highlighted in Appendix 2. **The Steering Committee (SC)**, whose members are responsible for planning and organising the study and scientific follow-up, includes the leaders of the six predefined work packages (WPs): WP1: Clinical phenotyping and biobanking (Professor Marte Lie Høivik), WP2: NORDTREAT trial (Professor Jonas Halfvarson), WP3: Host and gut microbiome signatures (NORDTREAT cohort)(Professor Vibeke Andersen), WP4: Bioinformatics and integrated analyses (Professor Dirk Repsilber), WP5: Exploitation and dissemination (Professor Johan D. Söderholm), and WP6: Project management (Professor Jonas Halfvarson). Elected SC members also include Professor Jens Kjeldsen, Professor Lóa G. Davíðsdóttir, Professor Einar S. Björnsson, Professor Asle W. Medhus, associate Professor Johannes R. Hov, consultant PhD Vendel Kristensen, associate Professor Randi Opheim, PhD Joachim Høg Mortensen, PhD fellow David Füchtbauer, and PhD fellow Anja Fejrskov. The Steering Committee conducts regular meetings and has feedback from the **External Advisory Board** composed of internationally renowned



For peer review only

representatives of academia and the biomedical industry: Professor Jack Satsangi, Oxford University (GB), Professor Philip Rosenstiel, Kiel University (DE), Professor Mauro D'Amato, Karolinska Institute (SE), vice president at Q-linea Mats Gullberg (SE), and from patient research partners (PRPs) in the national patient organizations (**Patient Advisory Council** [**Appendix 2**]). This project was built with input from the PRPs in the Danish Colitis-Crohn's Association, the Norwegian Mage-tarmforbundet, the Swedish Mag- och tarmförbundet, and from Icelandic patient representatives.

Finally, the **Executive Committee** (members listed in **Appendix 2**) is responsible for project coordination and management during the research project's preparation, operation and finalisation. Biweekly online meetings are conducted, ad-hoc meetings when necessary, and face-to-face meetings are held twice a year.

Perspectives

Substantial differences exist between patients suspected of IBD when comparing symptoms, onset, prognosis and treatment, which makes a one-size-fits-all approach insufficient. With current knowledge and clinical methodologies, the diagnosis and prognosis of IBD is challenging without invasive investigations. However, newly identified biomarkers may have implications for future disease management, as they could be used to differentiate patients with IBD and patients with other comparable gastrointestinal symptoms or predict disease outcomes. If we succeed in identifying diagnostic or prognostic biomarkers of IBD substantial improvement will be given to IBD treatment, including reduced side-effects and complications, increased quality of life for patients and their relatives and a reduced socioeconomic burden on society. Relevant findings with potential clinical value will be evaluated and replicated in other prospective cohorts if possible.

Dissemination of results to the public and scientifically



For peer review only - http://bmjopen.bmj.com/site/about/guidelines.xhtml

The target journal for the primary outcome will be a gastroenterology medical journal. Subsequently, other hypotheses will be tested, and manuscripts will be prepared with the intention of submitting to specialised journals in microbiome research, immunology, genetics, nutrition and gastroenterology. In addition to the scientific reporting, key findings with translational implications will be communicated to health professionals, patient organisations, public health policymakers, and the public through various media and news activities. When disseminating the results, we will apply the recommendations of the International Committee of Medical Journal Editors (ICMJE).[38]

For peer review only

Author affiliations

[1] Department of Medical Gastroenterology S, Odense University Hospital, Odense, Denmark

[2] Molecular Diagnostics and Clinical Research Unit, Institute of Regional Health Research, University Hospital of Southern Denmark, Hospital Sønderjylland, Aabenraa, Denmark

[3] Section for Biostatistics and Evidence-Based Research, the Parker Institute, Bispebjerg and Frederiksberg Hospital, Copenhagen, Denmark

[4] Internal Medicine & Emergency Department Svendborg, Odense University Hospital, Svendborg, Denmark

[5] Research Unit of Medical Gastroenterology, Department of Clinical Research, University of Southern Denmark, Odense, Denmark

[6] Department of Gastroenterology, Landspitali University Hospital, Reykjavik, Iceland

[7] Department of Internal Medicine, Örebro University Hospital, Region Örebro län, Örebro, Sweden

[8] Department of Gastroenterology, Oslo University Hospital, Oslo, Norway



For peer review only - http://bmjopen.bmj.com/site/about/guidelines.xhtml

[9] Department of Internal Medicine–Gastroenterology, University Hospital of Southern Denmark, Lillebaelt Hospital, Vejle, Denmark

[10] Gastrointestinal Diseases, Nordic Bioscience A/S, Herlev, Denmark

[11] Research Unit of Medical gastroenterology and hepatology, University Hospital of Southern Denmark, Hospital South West Jutland, Esbjerg, Denmark

[12] Jönköping Academy for Improvement in Health and Welfare, Jönköping University, Jönköping, Sweden

[13] Futurum-Academy for Healthcare, Region Jönköping County, Jönköping, Sweden

[14] School of Health and Medical Sciences, Örebro University, Örebro, Sweden

[15] Department of Biomedical and Clinical Sciences, Linköping University, Linköping, Sweden

[16] Institute of Regional Health Research, University of Southern Denmark, Odense, Denmark

[17] Institute of Molecular Medicine, University of Southern Denmark, Odense, Denmark

[18] OPEN - Open Patient data Explorative Network, University of Southern Denmark, Odense, Denmark

[19] Research Unit of Rheumatology, Department of Clinical Research, University of Southern Denmark, Odense University Hospital, Odense, Denmark

Acknowledgements

Acknowledgements are based on the ICMJE recommended criteria for authorship.[38]

Professor Björnsson ES, Professor Carlson M, Professor Heitmann BL, associate Professor Hov JR, CEO in Bio-Me Isaksen M, Professor Kruse TA, Professor Moum B, associate Professor Ophei



For peer review only - http://bmjopen.bmj.com/site/about/guidelines.xhtml

R, MD, PhD Ricanek P, and senior coordinator and innovation advisor Wallin Å contributed to the design of the study. Thanks to research advisor, MPH Caroline Moos for English text editing.

Authors' contributions (ORCID in parentheses)

Fejrskov A (0000-0002-1257-8630), Füchtbauer JD (0000-0002-9591-2963), Andersen V (0000-0002-0127-2863), Christensen R (0000-0002-6600-0631), and Kjeldsen J (0000-0001-8148-6572) wrote the first draft. Davíðsdóttir LG, Halfvarson J (0000-0003-0122-7234), Høivik ML (0000-0002-0104-465X), Mortensen JH (0000-0003-0326-7264), Rejler M (0000-0003-3286-2898), Repsilber D (0000-0002-7173-5579), Söderholm JD (0000-0002-3250-5367), Andersen V, Christensen R, and Kjeldsen J contributed to the conception and design of the study and are all members of the Executive Committee. Jensen MD (0009-0004-8432-1764), Nielsen LN, and Aalykke C are PIs and contributed to the inclusion of patients in the Region of Southern Denmark. All authors accepted the final submitted version.

Funding

The NORDTREAT project has received funding from NordForsk (30.000.000 SKR) via Vinnova (grant number 2019-01185 NORDTREAT), Rannis (NordForsk no. 90569 - Project no. 199782-0611), The Research Council of Norway (grant no. 2988039), and Innovation Fund Denmark (90569 NORDTREAT – grant number 8114-00026B). The Danish partners have allocated the funding (4.800.000 DKR) for remuneration of project staff, biobanking, and analyses of biological material. Furthermore, the University Hospital of Southern Denmark, Hospital Sønderjylland has funded 525.000DKR for a Ph.D. salary (Fejrskov A), The Region of Southern Denmark (Fri og Strategisk Forskning) has funded 1.137.000 DKR (Fejrskov A and Füchtbauer JD) for biobanking and analyses of biological material. Knud and Edith Eriksen Memorial Fund has funded 75.000 DKR for a Ph.D. salary (Fejrskov A). Odense University Hospital has funded 584.000 DKR for a Ph.D. salary (Füchtbauer JD). Section for Biostatistics and Evidence-Based Research, the Parker Institute, Bispebjerg and Frederiksberg Hospital (Christensen R) is



For peer review only

supported by a core grant from the Oak Foundation (OCAY-13-309). None of the researchers participating in the project have any financial connection to the funding organisation, nor do they have a financial interest in the study.

Competing interests

All authors declare no conflict of interest.

The funding sponsors had no role in the study's design; collection, management, analysis, or interpretation of data; in writing of the protocol, or the decision to submit results for publication.

Patient consent

Written informed consent will be obtained from participants after receiving verbal and written project information and before participating in the study.

Ethics approval

The Regional Scientific Ethical Committee (S-20200 051) and the Danish Data Protection Agency (20/54594) have approved the project. The procedures followed are in accordance with the ethical standards of the responsible committee on human experimentation (institutional and national) and with the Helsinki Declaration of 1975 with later amendments.

Provenance and peer review

Not commissioned; externally peer-reviewed.



For peer review only

Open Access

This is an Open Access article distributed in accordance with the Creative Commons Attribution Non Commercial (CC BY-NC 4.0) license (https://creativecommons.org/ licenses/by-nc/4.0/). This license permits others to share, redistribute, adapt, and build upon the work non-commercially, and license their derivative works on different terms, provided the original work is properly cited and the use is non-commercial.

For peer review only

REFERENCES


1 Zhao M, Gönczi L, Lakatos PL, *et al.* The Burden of Inflammatory Bowel Disease in Europe in 2020. *J Crohns Colitis*. 2021;15:1573–87.

2 Alatab S, Sepanlou SG, Ikuta K, *et al.* The global, regional, and national burden of inflammatory bowel disease in 195 countries and territories, 1990–2017: a systematic analysis for the Global Burden of Disease Study 2017. *Lancet Gastroenterol Hepatol*. 2020;5:17–30.

3 Ng SC, Shi HY, Hamidi N, *et al.* Worldwide incidence and prevalence of inflammatory bowel disease in the 21st century: a systematic review of population-based studies. *The Lancet*. 2017;390:2769–78.

4 Kaplan GG, Windsor JW. The four epidemiological stages in the global evolution of inflammatory bowel disease. *Nat Rev Gastroenterol Hepatol*. 2021;18:56–66. doi: 10.1038/s41575-020-00360-x

5 Maaser C, Sturm A, Vavricka SR, *et al.* ECCO-ESGAR Guideline for Diagnostic Assessment in IBD Part 1: Initial diagnosis, monitoring of known IBD, detection of complications. *J Crohns Colitis*. 2019;13:144-164K.

6 Binder V, Both H, Hansen PK, *et al.* Incidence and prevalence of ulcerative colitis and Crohn's disease in the County of Copenhagen, 1962 to 1978. *Gastroenterology*. 1982;83:563–8.

7 Lennard-Jones JE, Shivananda S. Clinical uniformity of inflammatory bowel disease a presentation and during the first year of disease in the north and south of Europe. EC-IBD Study Group. *Eur J Gastroenterol Hepatol*. 1997;9:353–9.

8 Schultze JL, Rosenstiel P. Systems Medicine in Chronic Inflammatory Diseases. *Immunity*. 2018;48:608–13.

9 Vicente AM, Ballensiefen W, Jönsson J-I. How personalised medicine will transform healthcare by 2030: the ICPerMed vision. *J Transl Med*. 2020;18:180.

10 Vision Paper on Personalised Medicine Research & Implementation by 2030 - ICPerMed. https://www.icpermed.eu/en/activities-vision-paper.php (accessed 13 September 2023)

11 Thorlacius-Ussing G, Schnack Nielsen B, Andersen V, *et al.* Expression and Localization of miR-21 and miR-126 in Mucosal Tissue from Patients with Inflammatory Bowel Disease. *Inflamm Bowel Dis*. 2017;23:739–52.




For peer review only - http://bmjopen.bmj.com/site/about/guidelines.xhtml

12 Zhou Y, Xu ZZ, He Y, *et al.* Gut Microbiota Offers Universal Biomarkers across Ethnicity in Inflammatory Bowel Disease Diagnosis and Infliximab Response Prediction. *mSystems*. 2018;3:e00188-17.

13 Mortensen JH, Manon-Jensen T, Jensen MD, *et al.* Ulcerative colitis, Crohn's disease, and irritable bowel syndrome have different profiles of extracellular matrix turnover, which also reflects disease activity in Crohn's disease. *PLoS ONE*. 2017;12:e0185855.

14 Bennike T, Birkelund S, Stensballe A, *et al.* Biomarkers in inflammatory bowel diseases: Current status and proteomics identification strategies. *World J Gastroenterol WJG*. 2014;20:3231–44.

15 van Rheenen PF, Van de Vijver E, Fidler V. Faecal calprotectin for screening of patients with suspected inflammatory bowel disease: diagnostic meta-analysis. *BMJ*. 2010;341:c3369.

16 Mortensen JH, Sinkeviciute D, Manon-Jensen T, *et al.* A Specific Calprotectin Neo-epitope [CPa9-HNE] in Serum from Inflammatory Bowel Disease Patients Is Associated with Neutrophil Activity and Endoscopic Severity. *J Crohns Colitis*. 2022;16:1447–60.

17 Mortensen JH, Godskesen LE, Jensen MD, *et al.* Fragments of Citrullinated and MMP-degraded Vimentin and MMP-degraded Type III Collagen Are Novel Serological Biomarkers to Differentiate Crohn's Disease from Ulcerative Colitis. *J Crohns Colitis*. 2015;9:863–72.

18 Schroeder KW, Tremaine WJ, Ilstrup DM. Coated Oral 5-Aminosalicylic Acid Therapy for Mildly to Moderately Active Ulcerative Colitis. *N Engl J Med*. 1987;317:1625–9.

19 Daperno M, D'Haens G, Van Assche G, *et al.* Development and validation of a new, simplified endoscopic activity score for Crohn's disease: the SES-CD. *Gastrointest Endosc*. 2004;60:505–12.

20 Silverberg MS, Satsangi J, Ahmad T, *et al.* Toward an Integrated Clinical, Molecular and Serological Classification of Inflammatory Bowel Disease: Report of a Working Party of the 2005 Montreal World Congress of Gastroenterology. *Can J Gastroenterol*. 2005;19:5A-36A.

21 Feng Y-S, Kohlmann T, Janssen MF, *et al.* Psychometric properties of the EQ-5D-5L: a systematic review of the literature. *Qual Life Res*. 2021;30:647–73.

22 Ware JE, Sherbourne CD. The MOS 36-Item Short-Form Health Survey (SF-36): I. Conceptual Framework and Item Selection. *Med Care*. 1992;30:473–83.

23 Guyatt G, Mitchell A, Irvine EJ, *et al.* A new measure of health status for clinical trials in inflammatory bowel disease. *Gastroenterology*. 1989;96:804–10.

24 Iwine EJ, Fagan BG, Wang' CJ. of a Quality of Life Index for Inflammatory Bowel Disease Change the Results? *Qual Life*.



For peer review only - http://bmjopen.bmj.com/site/about/guidelines.xhtml

25 Vestergaard C, Dahlerup JF, Bager P. Validation of the Danish version of Inflammatory Bowel Disease Self-assessment Scale. 2017.

26 Palsson OS, Whitehead WE, Van Tilburg MAL, *et al.* Development and Validation of the Rome IV Diagnostic Questionnaire for Adults. *Gastroenterology*. 2016;150:1481–91.

27 Harvey RF, Bradshaw JM. A SIMPLE INDEX OF CROHN'S-DISEASE ACTIVITY. *The Lancet*. 1980;315:514.

28 Walsh AJ, Bryant RV, Travis SPL. Current best practice for disease activity assessment in IBD. *Nat Rev Gastroenterol Hepatol*. 2016;13:567–79.

29 The Danish Health Examination Survey 2007-2008 (DANHES 2007-2008). https://doi.org/10.1177/1403494810393557

30 Maaser C, Sturm A, Vavricka SR, *et al.* ECCO-ESGAR Guideline for Diagnostic Assessment in IBD Part 1: Initial diagnosis, monitoring of known IBD, detection of complications. *J Crohns Colitis*. 2019;13:144-164K.

31 Turner D, Ricciuto A, Lewis A, *et al.* STRIDE-II: An Update on the Selecting Therapeutic Targets in Inflammatory Bowel Disease (STRIDE) Initiative of the International Organization for the Study of IBD (IOIBD): Determining Therapeutic Goals for Treat-to-Target strategies in IBD. *Gastroenterology*. 2021;160:1570–83.

32 Brodersen JB, Knudsen T, Kjeldsen J, *et al.* Diagnostic accuracy of pan-enteric capsule endoscopy and magnetic resonance enterocolonography in suspected Crohn's disease. *United Eur Gastroenterol J*. 2022;10:973–82.

33 Burisch J, Katsanos KH, Christodoulou DK, *et al.* Natural Disease Course of Ulcerative Colitis During the First Five Years of Follow-up in a European Population-based Inception Cohort—An Epi-IBD Study. *J Crohns Colitis*. 2019;13:198–208.

34 Burisch J, Andersen V, Čuković-Čavka S, *et al.* DOP019 Immunomodulators reduce the risk of surgery and hospitalisation in Crohn's disease in a prospective European population-based inception cohort: the Epi-IBD cohort. *J Crohns Colitis*. 2018;12:S042–3.

35 Bossuyt PM, Reitsma JB, Bruns DE, *et al.* STARD 2015: an updated list of essential items for reporting diagnostic accuracy studies. *BMJ*. 2015;351:h5527.

36 Collins GS, Reitsma JB, Altman DG, *et al.* Transparent reporting of a multivariable prediction model for individual prognosis or diagnosis (TRIPOD): the TRIPOD statement. *BMJ*. 2015;350:g7594.

37 von Elm E, Altman DG, Egger M, *et al.* Strengthening the reporting of observational studies in epidemiology (STROBE) statement: guidelines for reporting observational studies. *BMJ*. 2007;335:806–8.



For peer review only - http://bmjopen.bmj.com/site/about/guidelines.xhtml

38 icmje-recommendations.pdf. https://www.icmje.org/icmje-recommendations.pdf (accessed 4 January 2023)

For peer review only



For peer review only - http://bmjopen.bmj.com/site/about/guidelines.xhtml

**Appendix 1:** Inclusion sites in the NORDTREAT Cohort study

| Country | Hospital |
|---|---|
| DK | Odense University Hospital |
| DK | OUH Svendborg Hospital |
| DK | SLB Vejle Hospital |
| DK | SVS Esbjerg Hospital |
| IS | Landspitali Reykjavik |
| NO | Oslo University Hospital |
| NO | Vestfold Hospital |
| NO | Drammen Hospital |
| NO | Skien Hospital |
| SE | Örebro University Hospital |
| SE | Högland Hospital, Eksjö |

**Appendix 2**: The operational organisation of the **NORDTREAT** Cohort study (country responsibility in parenthesis)

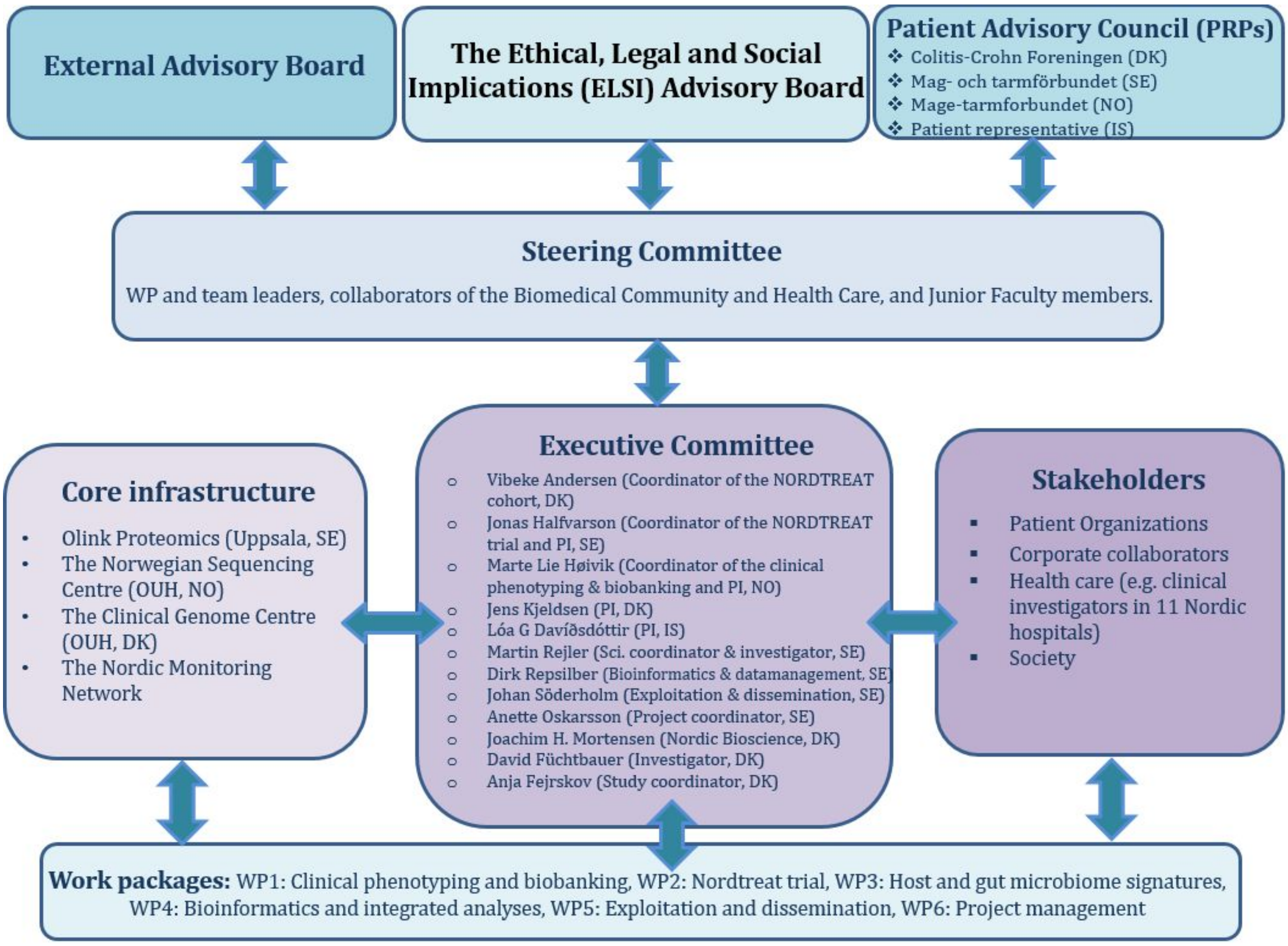


Abbreviations: WP: Work package, OUH, NO: Oslo University Hospital, Norway, OUH, DK: Odense University Hospital, Denmark, PI: Principal investigator, Sci.: Scientific, SE: Sweden and IS: Iceland

## STROBE 2007 (v4) Statement—Checklist of items that should be included in reports of *cohort studies*

| Section/Topic | Item # | Recommendation | Reported on page # |
|---|---|---|---|
| **Title and abstract** | 1 | (*a*) Indicate the study's design with a commonly used term in the title or the abstract | 1 |
| | | (*b*) Provide in the abstract an informative and balanced summary of what was done and what was found | 2 |
| **Introduction** | | | |
| Background/rationale | 2 | Explain the scientific background and rationale for the investigation being reported | 3-4 |
| Objectives | 3 | State specific objectives, including any prespecified hypotheses | 4-5 |
| **Methods** | | | |
| Study design | 4 | Present key elements of study design early in the paper | 5-7 |
| Setting | 5 | Describe the setting, locations, and relevant dates, including periods of recruitment, exposure, follow-up, and data collection | 7 |
| Participants | 6 | (*a*) Give the eligibility criteria, and the sources and methods of selection of participants. Describe methods of follow-up | 7-8 |
| | | (*b*) For matched studies, give matching criteria and number of exposed and unexposed | na |
| Variables | 7 | Clearly define all outcomes, exposures, predictors, potential confounders, and effect modifiers. Give diagnostic criteria, if applicable | 8-11 |
| Data sources/ measurement | 8* | For each variable of interest, give sources of data and details of methods of assessment (measurement). Describe comparability of assessment methods if there is more than one group | *8-11* |
| Bias | 9 | Describe any efforts to address potential sources of bias | na |
| Study size | 10 | Explain how the study size was arrived at | 13-14 |
| Quantitative variables | 11 | Explain how quantitative variables were handled in the analyses. If applicable, describe which groupings were chosen and why | na |
| Statistical methods | 12 | (*a*) Describe all statistical methods, including those used to control for confounding | 13-16 |
| | | (*b*) Describe any methods used to examine subgroups and interactions | na |
| | | (*c*) Explain how missing data were addressed | na |
| | | (*d*) If applicable, explain how loss to follow-up was addressed | na |
| | | (*e*) Describe any sensitivity analyses | na |
| **Results** | | | |

For peer review only

| | | | |
|---|---|---|---|
| Participants | 13* | (a) Report numbers of individuals at each stage of study—eg numbers potentially eligible, examined for eligibility, confirmed eligible, included in the study, completing follow-up, and analysed | na |
| | | (b) Give reasons for non-participation at each stage | na |
| | | (c) Consider use of a flow diagram | na |
| Descriptive data | 14* | (a) Give characteristics of study participants (eg demographic, clinical, social) and information on exposures and potential confounders | na |
| | | (b) Indicate number of participants with missing data for each variable of interest | na |
| | | (c) Summarise follow-up time (eg, average and total amount) | na |
| Outcome data | 15* | Report numbers of outcome events or summary measures over time | na |
| Main results | 16 | (*a*) Give unadjusted estimates and, if applicable, confounder-adjusted estimates and their precision (eg, 95% confidence interval). Make clear which confounders were adjusted for and why they were included | na |
| | | (*b*) Report category boundaries when continuous variables were categorized | na |
| | | (*c*) If relevant, consider translating estimates of relative risk into absolute risk for a meaningful time period | na |
| Other analyses | 17 | Report other analyses done—eg analyses of subgroups and interactions, and sensitivity analyses | na |
| **Discussion** | | | |
| Key results | 18 | Summarise key results with reference to study objectives | na |
| **Limitations** | | | |
| Interpretation | 20 | Give a cautious overall interpretation of results considering objectives, limitations, multiplicity of analyses, results from similar studies, and other relevant evidence | na |
| Generalisability | 21 | Discuss the generalisability (external validity) of the study results | na |
| **Other information** | | | |
| Funding | 22 | Give the source of funding and the role of the funders for the present study and, if applicable, for the original study on which the present article is based | 20-21 |

*Give information separately for cases and controls in case-control studies and, if applicable, for exposed and unexposed groups in cohort and cross-sectional studies.

**Note:** An Explanation and Elaboration article discusses each checklist item and gives methodological background and published examples of transparent reporting. The STROBE checklist is best used in conjunction with this article (freely available on the Web sites of PLoS Medicine at http://www.plosmedicine.org/, Annals of Internal Medicine at http://www.annals.org/, and Epidemiology at http://www.epidem.com/). Information on the STROBE Initiative is available at www.strobe-statement.org.

For peer review only

BMJ Open

# Novel Biomarker Profiles to Improve Individual Diagnosis and Prognosis in Patients with Suspected Inflammatory Bowel Disease: Protocol for the Nordic Inception Cohort Study (NORDTREAT)

| | |
|---|---|
| Journal: | *BMJ Open* |
| Manuscript ID | bmjopen-2023-083144.R1 |
| Article Type: | Protocol |
| Date Submitted by the Author: | 08-Apr-2024 |
| Complete List of Authors: | Fejrskov, Anja; Odense University Hospital, Department of Medical Gastroenterology S; University of Southern Denmark Faculty of Health Sciences, Molecular Diagnostics and Clinical Research Unit<br>Füchtbauer, Johannes David; Odense University Hospital, Department of Medical Gastroenterology S; University of Southern Denmark, Research Unit of Medical Gastroenterology, Department of Clinical Research<br>Davíðsdóttir, Lóa ; Landspitali National University Hospital of Iceland, Department of Gastroenterology<br>Halfvarson, Jonas; Örebro University Hospital, Department of Internal Medicine<br>Høivik, Marte ; Oslo University Hospital, Department of Gastroenterology; University of Oslo Institute for Clinical Medicine<br>Jensen, Michael ; Lillebaelt Hospital - University Hospital of Southern Denmark, Department of Internal Medicine–Gastroenterology; University of Southern Denmark Faculty of Health Sciences, Institute of Regional Health Research<br>Mortensen, Joachim ; Nordic Bioscience A/S, Gastrointestinal Diseases<br>Nielsen, Lene; Hospital South West Jutland, Research Unit of Medical gastroenterology and hepatology<br>Rejler, Martin; Jönköping University, Jönköping Academy for Improvement in Health and Welfare; Futurum Academy of Health and Care, Futurum-Academy for Healthcare<br>Repsilber, Dirk; Örebro University, School of Health and Medical Sciences<br>Söderholm, Johan; Linköping University, Department of Biomedical and Clinical Sciences<br>Aalykke, Claus; Odense University Hospital, Internal Medicine and Emergency Department<br>Andersen, Vibeke; University of Southern Denmark Faculty of Health Sciences, Molecular Diagnostics and Clinical Research Unit; University of Southern Denmark, Institute of Molecular Medicine<br>Christensen, Robin; Parker Instituttet, Section for Biostatistics and Evidence-Based Research; University of Southern Denmark, Research Unit of Rheumatology, Department of Clinical Research<br>Kjeldsen, Jens; Odense University Hospital, Department of Medical Gastroenterology S; University of Southern Denmark, Research Unit of Medical Gastroenterology, Department of Clinical Research |
| <b>Primary Subject | Gastroenterology and hepatology |

| | |
|---|---|
| Heading</b>: | |
| Secondary Subject Heading: | Diagnostics |
| Keywords: | Inflammatory bowel disease < GASTROENTEROLOGY, Prognosis, Gastroenterology < INTERNAL MEDICINE, Endoscopy < GASTROENTEROLOGY, Adult gastroenterology < GASTROENTEROLOGY |
| | |


SCHOLARONE™
Manuscripts

# Novel Biomarker Profiles to Improve Individual Diagnosis and Prognosis in Patients with Suspected Inflammatory Bowel Disease: Protocol for the Nordic Inception Cohort Study (NORDTREAT)

**Authors:** Fejrskov A [1, 2, 3, *], Füchtbauer JD [1, 4, 5, *], Davíðsdóttir LG [6], Halfvarson J [7], Høivik ML [8, 20], Jensen MD [9, 16], Mortensen JH [10], Nielsen LN [11], Rejler M [12, 13], Repsilber D [14], Söderholm JD [15], Aalykke C [4], Andersen V [2, 16, 17, 18, °], Christensen R [3, 19, °], Kjeldsen J [1, 5, °]

* Share first authorship ° Share last authorship

For numbered affiliations and open researcher and contributor ID (ORCID), see end of article.

**Correspondence to:** PhD fellow Anja Fejrskov; Anja.Fejrskov@rsyd.dk; PhD fellow Johannes David Füchtbauer; David.Fuchtbauer@rsyd.dk

**Key words:** IBD, inflammatory bowel disease, ulcerative colitis, Crohn’s disease, biomarkers, diagnosis, prognosis, personalised medicine.



For peer review only

ABSTRACT

**Introduction.** Inflammatory bowel disease (IBD), including ulcerative colitis and Crohn´s disease, can be challenging to diagnose, and treatment outcomes are difficult to predict. In the NORDTREAT cohort study, a Nordic prospective multicentre study, we aim to identify novel molecular biomarkers of diagnostic value by assessing the diagnostic test accuracy (cross-sectionally), as well as the prognostic utility when used as prognostic markers in the long-term (cohort study). In the diagnostic test accuracy study, the primary outcome is a successful diagnosis using one or more novel index tests at baseline compared to the ECCO criteria as the reference standard. The composite outcome of the prognostic utility study is "severe IBD" within 52 weeks from inclusion, defined as one or more of the following three events: IBD-related surgery, IBD-related hospitalisation or IBD-related death.

**Methods and analysis.** We aim to recruit 800 patients referred on suspicion of IBD to this longitudinal observational study, a collaboration between eleven inclusion sites in Denmark, Iceland, Norway, and Sweden. Inclusion will occur from February 2022 until December 2023 with screening and baseline visits for all participants and three outcome visits at weeks 12, 26 and 52 after baseline for IBD-diagnosed patients. Biological material (blood, faeces, biopsies, urine, and hair), clinical data, and lifestyle information will be collected during these scheduled visits.

**Ethics and dissemination.** This study will explore novel biomarkers to improve diagnostic accuracy and prediction of disease progression, thereby improving medical therapy and the quality of life for patients with IBD.

The study is approved by the Ethics Committee (DK: S-20200051, v1.4, 16.10.2021; IS: VSNb2021070006/03.01, NO: 193064; SE: DNR 2021-05090) and the Danish Data Protecting Agency (20/54594). Results will be disseminated through peer-reviewed journals, patient associations and presentations at international conferences.

Clinical trial registration number NCT05414578.




For peer review only

Strengths and limitations of this study

- All participants included on suspicion of IBD and treatment naïve
- Strong international collaboration and extensive inclusion area with 11 sites in four Nordic countries
- Prospective, longitudinal study design with rigorously collected biological material and data on disease activity and lifestyle
- Patient heterogeneity (four countries)
- Ascertainment bias; as some patients with severe disease course may not be eligible for inclusion if acute treatment is initiated

INTRODUCTION

Inflammatory bowel disease (IBD), primarily ulcerative colitis (UC) and Crohn´s disease (CD) are idiopathic disorders characterised by chronic gastrointestinal inflammation and relapsing symptoms like diarrhoea, abdominal pain, and fatigue. A large proportion of the patients experience severe disease manifestations like bowel obstruction, fistulising disease, liver disease, and colorectal cancer. Additionally, extra-intestinal manifestations occur in 15-40%.[1] Consequently, hospitalisations, surgical interventions, recurring sick leave and life-long medication severely impact the lives of patients and families. Even though the incidence of IBD appears to have reached a plateau in most Western European countries, it is rapidly increasing in many newly industrialised countries.[2] The prevalence of IBD has increased substantially in the last three decades, exceeding 0.5 % in many European countries.[2–4] An estimated 1.3 million people in Europe suffer from IBD, imposing a substantial health, social, and economic burden.[1] There is a need to improve the management of IBD.



For peer review only - http://bmjopen.bmj.com/site/about/guidelines.xhtml

Diagnosis of IBD can be cumbersome.[5–7] Furthermore, IBD is a heterogeneous disease and its course differs substantially between patients. The lack of vigorous biomarkers for individual disease characterisation potentially leads to delayed diagnosis, organ damage, worsened outcome, increased mortality, and an ever-increasing disease burden.[8] Personalised medicine (PM) could be a suitable strategy for optimising both diagnostics and treatment of IBD.[9] The vision of the International Consortium for Personalised Medicine (ICPerMed) describes "*an approach to medicine that integrates an individual's characteristics for early disease diagnosis, prognosis, optimal choice of treatment, accurate disease risk estimation, and targeted prevention*".[10] In gastrointestinal diseases, PM is based upon advancements in biomarker detection in a variety of omics-platforms, including genomics, transcriptomics, microbiomics, proteomics, and metabolomics, as well as body imaging methods, e.g., computed tomography (CT) scans, and magnetic resonance imaging (MRI) scans. Recent advances led to the identification of several potential biomarkers for diagnosis and treatment response in IBD.[11–17] However, they are not in use in clinical practice. Even though early diagnosis and efficient treatment strategies for IBD are cornerstones for reducing irreversible organ damage, identifying biomarkers that can efficiently support clinical decision-making is still warranted.

This protocol article introduces a prospective clinical study that aims to identify novel diagnostic biomarkers for more accurate early diagnosis of IBD and assess the utility of novel prognostic biomarkers in clinical outcomes with various disease manifestations. Notably, the study evaluates biomarkers in a relevant clinical setting, i.e. among patients referred to the hospital on suspicion of IBD and diagnosed according to the ECCO Criteria.[5]

For peer review only

## Aims

This study aims, on the one hand, to improve accuracy in diagnosing IBD in the form of a *diagnostic test accuracy study*. The main hypothesis is that biochemical markers can differentiate individuals referred to a gastroenterology department with suspected IBD into individuals with (IBD) and without (non-IBD) diagnosis. The primary aim is to identify molecular biomarkers (e.g., from blood and stools) for discriminating between individuals diagnosed with IBD (CD, UC, and unclassified inflammatory bowel disease [IBD-U]) and non-

IBD. Secondary aims include investigating whether clinical, genetic, microbiome and lifestyle information and combinations thereof (e.g., gene-environment interaction analyses) are associated with IBD diagnosis.

On the other hand, the main hypothesis of the *prognostic utility study* is that prognostic factors can distinguish different pathways of disease progression. The primary aim of this study is to identify prognostic biomarkers that can define different IBD disease courses. The secondary aims are to evaluate whether including information on clinical and lifestyle factors and combinations thereof can improve prognostic value.

## METHODS and ANALYSES

### Design

The NORDTREAT cohort study is a Nordic multicentre collaboration on diagnostic factors and prognostic factors of IBD among patients referred to the hospital with suspected IBD. All referred patients will be carefully evaluated to ensure that patients with suspected IBD are invited to participate in the study at their first clinic visit. Informed consent will be obtained at the screening visit (week -4). After study material collection (i.e. blood, stool, biopsies, hair, urine, questionnaires, and patient-reported outcome measures [PROMs]) and diagnostic evaluation, IBD diagnosis will be established or rejected at the baseline visit (week 0). IBD patients will be followed until week 52 with three additional visits (week 12, 26, and 52), whereas non-IBD patients will complete questionnaires at baseline visit and week 52 (**Figure 1**).

**Figure 1:**

The prospective study consists of a cross-sectional part with focus on diagnosis and a longitudinal cohort study (52 weeks) with focus on prognosis (i.e., a prognostic utility study).



For peer review only - http://bmjopen.bmj.com/site/about/guidelines.xhtml

The inclusion of patients into the NORDTREAT cohort study will be reported as a flowchart (**Figure 2**).

The objective of the *diagnostic test accuracy study* is to determine the sensitivity and specificity of biomarkers of interest (baseline measurements, see Biomarkers section) to diagnose IBD when applying the ECCO Diagnostic criteria as the reference standard.[5]

The objective of the *prognostic utility study* is to examine whether the biomarkers of interest predict severe disease outcomes in patients with IBD. Severe IBD is defined as those experiencing IBD-related surgery, IBD-related hospitalisation (except planned procedures), or IBD-related death within 52 weeks of inclusion.

**Figure 2:**

Setting

This Nordic multicentre study is a collaboration between gastroenterology departments in 11 Nordic hospitals (**Appendix 1**). The study enrolment will occur between 7 February 2022 and 31 December 2023. The first patient's first visit (FPFV) was conducted in Oslo University Hospital in February 2022, and the first patient's last visit (FPLV) was in February 2023. All inclusion sites will conduct their last patient's first visit (LPFV) and last patient's last visit (LPLV) in December 2023 and December 2024, respectively.

Patient characteristics and eligibility criteria

Inclusion criteria: Patients ≥18 years of age referred to a gastroenterology department with suspected IBD who give written informed consent to participate in the study. Exclusion criteria: Patients with a previous diagnosis of CD, UC or IBD-U or patients unable to provide informed consent or comply with protocol requirements, e.g. due to recreational drug or alcohol misuse.



For peer review only

The clinical data and lifestyle information collected for the study will be presented in tabular form (see **Table 1**) and include:

**Clinical interview:** Demographics (sex, age, marital status, educational level, occupational status, and smoking), disease history (previous disease including debut of gastrointestinal symptoms) and surgery (abdominal and perianal), occurrence of intestinal complications (defined as strictures and fistulas), occurrence of perianal disease, extra-intestinal manifestations, medication (past and present), family history (of IBD and colorectal cancer) and at the week 52 visit: relapses and surgery (abdominal and perianal) are recorded.

**Clinical examination:** includes a perianal examination of study subjects with perianal CD (accounting for the number of draining and non-draining perianal fistula).

**Vital signs:** include measurement of height, weight, heart rate, blood pressure, and temperature.

**Endoscopy:** Standardised scoring systems will record macroscopic findings from the ileocolonoscopy: For patients with UC, the Endoscopic Mayo Score per segment is applied, i.e. ascending-, transverse-, descending-, sigmoid colon and rectum.[18] For patients with CD, the Simple Endoscopic Score for Crohn's Disease (SES-CD) per segment is applied, i.e. terminal ileum, ascending-, transverse-, left colon and rectum.[19] Patients diagnosed with IBD-U at baseline will be assessed with the Endoscopic Mayo Score per segment and the SES-CD per segment. The endoscopy is recorded on video (approximately 20 seconds per segment). Furthermore, standard routine biopsies from the ileum, ascending-, transverse-, descending colon, and rectum will be obtained.

**Medical records:** Routine clinical information, clinical findings and procedures (endoscopic and surgical), test results (i.e. histology, radiology [including small bowel MRI, capsule endoscopy, CT scan, and ultrasonography], microbiology, and biochemistry), diagnosis, treatment and complications including hospitalisations will be retrieved from the medical records.



For peer review only - http://bmjopen.bmj.com/site/about/guidelines.xhtml

**Montreal classification:** Based on results from the routine examinations, the study subjects will be classified according to the internationally accepted IBD classification system, the Montreal Classification.[20]

**Lifestyle information, Disease activity index, Disease questionnaires and PROMs:** Participants will complete validated questionnaires (on paper or electronically) on disease activity, quality of life, including **EQ-5D-5L**[21] (measure of e.g. mobility, personal care, usual activities, pain/discomfort, and anxiety/depression), **SF-36**[22] (including perception of health, limitations in daily and social activities, general mental health [psychological distress and well-being], and vitality [energy and fatigue] within the last 4 weeks), **IBDQ**[23,24] (disease impact on patients daily functioning and quality of life e.g. abdominal pain, sleep, incontinence, ability to work, and conduct social activities within the last 2 weeks), **IBD-F**[25] (disease specific questionnaire comprising five questions about the severity of fatigue [Part 1] and **ROME IV**[26] (The Rome IV Diagnostic Questionnaire for Functional Gastrointestinal Disorders in Adults). The electronic case report form (eCRF) platform Viedoc™ (Viedoc Technologies, Uppsala Sweden) is applied, and in Denmark REDCap (Vanderbilt University, Nashville, TN, USA) is used for diet questionnaires.

Before each visit, patients with IBD will register clinical disease activity, Harvey-Bradshaw Index (**HBI**)[27,28] (diary for patients with CD including two clinical parameters: abdominal pain and number of liquid or soft stools), or **Partial Mayo Score** (diary for patients with UC or IBD-U including the two non-invasive components of the full Mayo Score [stool frequency and rectal bleeding]). All participants will perform diet questionnaires at baseline and week 52.[29]

For peer review only

**Table 1.** Overview of clinical and lifestyle information collected according to visits (weeks after diagnosis)

| | IBD | | | | non-IBD | |
|---|---|---|---|---|---|---|
| Week | - 4 / 0 | 12 | 26 | 52 | - 4 /0 | 52 |



For peer review only - http://bmjopen.bmj.com/site/about/guidelines.xhtml

| | | | | | | |
|---|---|---|---|---|---|---|
| Clinical information | | | | | | |
| Clinical interview and examination | x | x | | x | x | |
| Vital signs | x | x | x | x | x | |
| Endoscopy | x | | | x | x | |
| Medical records | x | x | | x | x | |
| Montreal classification | x | | | x | | |
| Lifestyle information, Disease activity index and PROMs | | | | | | |
| SF36 | x | x | x | x | | |
| IBDQ | x | x | x | x | | |
| EQ-5D-5L | x | x | x | x | | |
| IBD-F | x | x | x | x | x | x |
| HBI (CD only) | x | x | x | x | | |
| Partial Mayo score (UC only) | x | x | x | x | | |
| ROME IV | x | x | x | x | x | x |
| Diet | x | | | x | x | x |

Abbreviations: SF36: Short Form 36, IBDQ: Inflammatory Bowel Disease Questionnaire, IBD-F: Inflammatory Bowel Disease Fatigue (Part 1) and HBI: Harvey-Bradshaw Index.

**Biological material** (blood, biopsies, faeces, hair and urine) will be collected at the scheduled visits (**Table 2**):

At baseline, all study subjects have blood samples (whole blood [4ml], plasma [10ml], serum [20ml] and Paxgene blood RNA system [2,5ml]) and biopsies collected (8 biopsies from predefined locations collected in Allprotect reagent and 6 biopsies from predefined locations snap frozen in liquid nitrogen or FlashFREEZE [Milestone Medical, product no. 100780 from Axlab A/S, DK], ensuring a standardised freezing procedure at -80°C). Furthermore, the faecal samples collected at the patient's home will consist of: one stool collection tube with RNALater, DNA stabiliser, and a dry sample, respectively, as well as a tuft of hair and urine (8ml).



For peer review only - http://bmjopen.bmj.com/site/about/guidelines.xhtml

Blood samples will not be collected on the day of the ileocolonoscopy, and faecal samples will not be collected during the colonic cleansing to minimise any potential influence from the standard cleansing procedure conducted before an endoscopy.

On follow-up visits at week 12 and week 26 all patients diagnosed with IBD will have blood samples (plasma [10ml] and serum [20ml]) and faecal samples (one stool collection tube with RNALater, DNA stabiliser and a dry sample, respectively) collected. At the week 52 visit for IBD patients, biological material similar to baseline will be collected (except for whole blood). The biological material will be stored at local facilities according to national procedures, and accordingly available for future research projects upon approval of the NORDTREAT executive committee. Standard operating procedures (SOPs) with detailed information can be found in **Appendix 2**.

**Table 2:** Overview of the biological material collected in the study

| Biological material | Specifications | Baseline<br>IBD and non-IBD | Week 12<br>IBD | Week 26<br>IBD | Week 52<br>IBD |
|---|---|---|---|---|---|
| Blood | Whole blood (EDTA-K2) | x | | | |
| | Plasma (EDTA-K2 ) | x | x | x | x |
| | Serum | x | x | x | x |
| | Paxgene | x | | | x |
| Biopsies | Allprotect reagent | x | | | x |
| | Snap frozen | x | | | x |
| Faecal samples | DNA stabiliser | x | x | x | x |
| | Dry | x | x | x | x |
| | RNALater | x | x | x | x |
| Hair | 10-20mg | x | | | x |
| Urine | Spot urine | x | | | x |

Primary endpoint(s)



For peer review only - http://bmjopen.bmj.com/site/about/guidelines.xhtml

The primary endpoint for the cross-sectional *diagnostic test accuracy study* is diagnosis according to the reference standard from ECCO criteria,[30] i.e. IBD (including CD, UC, IBD-U) or non-IBD (patients not diagnosed with IBD) at referral.

The primary endpoint for the longitudinal *prognostic utility study* is a composite outcome measure. We will include multiple (three mutually associated) endpoint components combined as a Boolean function; this will be our primary outcome measure derived from randomised trials while also anticipating that the approach will be associated with increased statistical efficiency (e.g. power). The composite outcome consists of the proportion of patients who experience severe IBD over 52 weeks after inclusion, defined as either IBD-related surgery, IBD-related hospitalisation (except planned procedures) or IBD-related death.

Key secondary endpoints in the prognostic utility study

Major secondary outcomes assessed and analysed 52 weeks from baseline will include IBD-related surgery (number [no.] in %), IBD-related hospitalisation (no., %), IBD-related deaths (no., %) as well as non corticosteroid-free (local acting systemic administered [budesonide] and systemic steroids [prednisolone]) clinical remission at week 12.[31] Clinical remission in CD is defined as patient-reported outcome 2 (abdominal pain ≤ 1 and stool frequency ≤ 3) or Harvey Bradshaw Index < 5. Clinical remission in UC is defined as patient-reported outcome 2 (rectal bleeding = 0 and stool frequency = 0) or Partial Mayo score (< 3 and no score >1). Non corticosteroid-free endoscopic remission is assessed at week 52[31] where endoscopic remission in CD is defined as Simple endoscopic score < 3 points or absence of ulcerations (e.g. SES-CD ulceration subscore = 0). Endoscopic remission in UC is defined as Mayo endoscopic score = 0 points or UC endoscopic index of severity ≤ 1 point. Disease impact on patient's life will be assessed by the 32-item version of Inflammatory Bowel Disease Questionnaire (IBDQ) score (number [%] ≤ 170) and the numerical change of the SF36 subscales.

Other secondary endpoints in the prognostic utility study



For peer review only - http://bmjopen.bmj.com/site/about/guidelines.xhtml

The composite secondary outcome is the proportion of patients who experience IBD complications within 52 weeks after inclusion, defined as the cumulative number of patients who experience one of the following (*i*) need for medication (immunomodulators, biologics or steroids) between diagnosis and one year, (*ii*) need for surgery between diagnosis and one year, (*iii*) need for hospitalisation between diagnosis and one year or (*iv*) relapse between diagnosis and one year. Furthermore, the cumulative number of flares, adverse drug reactions, numerical change in the Fatigue Questionnaire, and cumulative glucocorticoid exposure will be assessed. Faecal calprotectin > 250 µg/g (no., %), time to start of biological therapy, time to IBD-related surgery or hospitalisation, and CD-related surgery (stenosis) will also be measured.

Mid-term and long-term complications in the prognostic utility study

IBD-related hospitalisations will be assessed and defined as a composite endpoint combining the total number of IBD-related hospitalisations (including re-admissions) and the cumulative length of hospital stay. Bowel damage in patients with CD (Lémann index for patients with an MRI), UC-related surgery (colectomy), CD-related surgery (no., %), IBD-related hospitalisations (no., %), and change in disease extent in UC (with a focus on macroscopic proximal disease extent) will be assessed among the mid-term complications. Among the long-term complications, the following three endpoints will be measured: gastrointestinal and extra-intestinal dysplasia (no., %), cancer (gastrointestinal and others in no., %), and mortality (no., %).

Biomarkers

At the time of submission, the NORDTREAT executive committee has approved projects on the following topics in relation to diagnosis and prognosis (including inflammatory intensity, disease extend, tissue damage, tissue repair activity, severe disease course): extra cellular matrix (ECM) proteins, microbiome, mycobiome, faecal markers, genetic risk factors, metabolomics anti-bodies towards microbial and auto-antigens.



For peer review only - http://bmjopen.bmj.com/site/about/guidelines.xhtml

Statistical methods

Referring to earlier studies, approximately 40% of a population referred with suspected IBD will have the diagnosis confirmed according to the ECCO criteria (or comparable).[5,32] Cost and practical issues will restrict the sample size to approximately 800 patients, with an expected 80% participation rate.

In the *diagnostic test accuracy study*, we will compare two independent binomial proportions, using the likelihood ratio statistic with a Chi-square approximation and two-sided significance level (0.05). With an expected sample size of 640 participants (assuming a biomarker positivity-to-negativity ratio of 1 to 1) we will reach an approximate statistical power of 100%, assuming the proportion of patients diagnosed with IBD is 80% in the biomarker positive group and 20% in the biomarker negative group. If we include 640 study participants, we will potentially ("rule of thumb") be able to build a multivariable statistical model with as many as 64 covariates. Thus, we will examine a variety of biochemical variables for diagnosing IBD (see Biomarkers section). The expectation is that a proportion of 100-200 preliminary biomarkers will be evaluated in this model to distinguish non-diagnostic markers and preliminary biomarkers that can be replicated. When applying a significance level of 0.05, each biomarker has a 5% risk of being replicated by chance. Thus, in the evaluation of, e.g. 100-200 biomarkers, five to ten biomarkers may be replicated by chance. In contrast, the risk of not replicating "true" biomarkers with low effects is estimated to be approximately 20%. All replicated biomarkers will be built into a prediction model that will be optimised and evaluated by cross-validation. The primary hypothesis is that the biomarker signature will be able to characterise the participant population into an IBD and a non-IBD group.

In the *prognostic utility study*, we expect a sample size of 256 IBD patients and a 10% event rate for severe IBD within 52 weeks.[33,34] If we assume a 15% event rate for the biomarker positive group and a 5% event rate for the biomarker negative group, and anticipate an even 1:1 biomarker positive-to-negative distribution, approximately 80 patients in each group will increase the statistical power above the 90% threshold.

**Comparison of measures of diagnostic accuracy:** The basic measures of quantifying the diagnostic accuracy of a test will be the diagnostic Odds Ratio, sensitivity, and specificity



For peer review only - http://bmjopen.bmj.com/site/about/guidelines.xhtml

measures. From these measures, we will calculate the likelihood ratio (LR), the ratio of the probability of the index test result among patients who have IBD to the probability of the same test among patients who do not have IBD (non-IBD). Since all diagnostic tests will be performed on each patient, paired data results and methods that account for the correlated binary outcomes are necessary (e.g., McNemar's test).

**Prognostic factor research (the prognostic utility study):** The primary statistical analysis will be based on the proportion of participants experiencing severe IBD among biomarker-positive versus biomarker-negative patients. The efficacy of the prognostic biomarker(s) will be considered as the proportion of the patients with different disease outcomes among the biomarker-positive patients compared to the biomarker-negative patients. The associations of the suspected important molecular biomarkers with other variables will be tested with nonparametric tests: with Spearman's rank correlation (rs) for continuous variables and the Wilcoxon rank-sum test or Kruskal-Wallis test, including a Wilcoxon-type test for trend across ordered groups where appropriate, for categorical variables.

Logistic regression models will be utilized with individual markers as the exposure variables and composite clinical severity as the outcome (dependent variable). The analyses will be adjusted simultaneously for sex, age, and prescribed therapy. Potential interactions between biomarker status (positive/negative) and specific drug type will be evaluated too. Covariates comprise of various measures within genetics, transcriptomics, microbiomes, and proteomics (see Biomarkers section). We will attempt to develop prediction models containing several conventional risk factors (i.e., age, sex, type of drug therapy) with or without any novel biomarkers and calculate improvements in predictive ability using risk discrimination and reclassification measures. We will use a 2-stage approach that allows for the examination of centre heterogeneity through the calculation of the C-index, a measure of risk discrimination, and changes within each centre before pooling results.

We will attempt to develop multivariable prediction models, relating multiple predictors for a particular individual to the probability of or risk for the presence (diagnosis of IBD at baseline) or future occurrence (prognosis) of a particular outcome, such as intestinal surgery within the first year. Predictors such as biomarkers are covariates, explored as prognostic or predictive



For peer review only - http://bmjopen.bmj.com/site/about/guidelines.xhtml

factors. Models will be built using regularised regression methods, such as sparse PLS or LASSO, as well as tree-based machine learning approaches, such as random forest, or non-linear non-symbolic deep learning approaches, capitalising on available published datasets. Also, multiple datasets will be used for integrative analyses, to explore if combinations or interactions of variables of different kinds could aid predictive or prognostic potential of our models.

Patient and public involvement

The NORDTREAT project has evolved in collaboration with patient research partners (PRPs) from the Danish Colitis-Crohn's Association, the Norwegian Mage-tarmforbundet, the Swedish Mag- och tarmförbundet, and from Icelandic patient representatives ensuring implementation of patient perspectives in aspects as e.g. research aims and outcomes. The PRPs were involved before the formal project start (adding value in the design of the study) and they are continuously informed on study progress. Overall the PRPs add value in the project conduct through their engagement in the Patient Advisory Council (**Appendix 3**) increasing knowledge-sharing, awareness on the project and research comprehensiveness.

When reporting the *diagnostic test accuracy study*, we will adhere to the STARD guideline[35] and the TRIPOD statement,[36] and when reporting the *prognostic utility study* we will apply the STROBE[37] as well as the TRIPOD statement.[36] Before any analyses are initiated an elaborated statistical analysis plan (SAP) indicating explicit figures and tables will be provided.

Project organisation

The NORDTREAT partnership builds on extensive professional experience, a strong track record of collaborative clinical projects and participation in a number of European and global consortia (e.g., the IBD Character Consortium, the SYSCID Consortium and the International IBD Genetic Consortium).

The NORDTREAT cohort study is conducted in parallel and as part of the NORDTREAT trial (a biomarker-stratified randomised controlled trial [RCT]). The aim of the NORDTREAT RCT is to



For peer review only - http://bmjopen.bmj.com/site/about/guidelines.xhtml

demonstrate that personalised therapy can be applied to patients with IBD, by assessing if a top-down treatment can improve clinical outcomes in IBD patients with an increased risk of poor disease course (https://clinicaltrials.gov/ct2/show/NCT05180175).

The operational organization of the NORDTREAT study is highlighted in **Appendix 3**. The **Steering Committee (SC)**, whose members are responsible for planning and organising the study and scientific follow-up, includes the leaders of the six predefined work packages (WPs): WP1: Clinical phenotyping and biobanking (Professor Marte Lie Høivik), WP2: NORDTREAT trial (Professor Jonas Halfvarson), WP3: Host and gut microbiome signatures (NORDTREAT cohort)(Professor Vibeke Andersen), WP4: Bioinformatics and integrated analyses (Professor Dirk Repsilber), WP5: Exploitation and dissemination (Professor Johan D. Söderholm), and WP6: Project management (Professor Jonas Halfvarson). Elected SC members also include Professor Jens Kjeldsen, Professor Lóa G. Davíðsdóttir, Professor Einar S. Björnsson, Professor Asle W. Medhus, associate Professor Johannes R. Hov, consultant PhD Vendel Kristensen, associate Professor Randi Opheim, PhD Joachim Høg Mortensen, PhD fellow David Füchtbauer, and PhD fellow Anja Fejrskov. The Steering Committee conducts regular meetings and has feedback from the **External Advisory Board** composed of internationally renowned representatives of academia and the biomedical industry: Professor Jack Satsangi, Oxford University (GB), Professor Philip Rosenstiel, Kiel University (DE), Professor Mauro D'Amato, Karolinska Institute (SE), vice president at Q-linea Mats Gullberg (SE), and from PRPs in the national patient organizations (**Patient Advisory Council** [**Appendix 3**]).

Finally, the **Executive Committee** (members listed in **Appendix 3**) is responsible for project coordination and management during the research project's preparation, operation and finalisation. Biweekly online meetings are conducted, ad-hoc meetings when necessary, and face-to-face meetings are held twice a year.

Perspectives

Substantial differences exist between patients suspected of IBD when comparing symptoms, onset, prognosis and treatment, which makes a one-size-fits-all approach insufficient. With



For peer review only - http://bmjopen.bmj.com/site/about/guidelines.xhtml

current knowledge and clinical methodologies, the diagnosis and prognosis of IBD is challenging without invasive investigations. However, newly identified biomarkers may have implications for future disease management, as they could be used to differentiate patients with IBD and patients with other comparable gastrointestinal symptoms or predict disease outcomes. If we succeed in identifying diagnostic or prognostic biomarkers of IBD substantial improvement will be given to IBD treatment, including reduced side-effects and complications, increased quality of life for patients and their relatives and a reduced socioeconomic burden on society. Relevant findings with potential clinical value will be evaluated and replicated in other prospective cohorts if possible.

## Ethics and dissemination

The study is approved by the Ethics Committee (DK: S-20200051, v1.4, 16.10.2021; IS: VSNb2021070006/03.01, NO: 193064; SE: DNR 2021-05090) and the Danish Data Protecting Agency (20/54594). Clinical trial registration number NCT05414578. The procedures followed are in accordance with the ethical standards of the responsible committee on human experimentation (institutional and national) and with the Helsinki Declaration of 1975 with later amendments.

The target journal for the primary outcome will be a gastroenterology medical journal. Subsequently, other hypotheses will be tested, and manuscripts will be prepared with the intention of submitting to specialised journals in microbiome research, immunology, genetics, nutrition and gastroenterology. In addition to the scientific reporting, key findings with translational implications will be communicated to health professionals, patient organisations, public health policymakers, and the public through various media and news activities. When disseminating the results, we will apply the recommendations of the International Committee of Medical Journal Editors (ICMJE).[38]



For peer review only - http://bmjopen.bmj.com/site/about/guidelines.xhtml
For peer review only

Author affiliations

[1] Department of Medical Gastroenterology S, Odense University Hospital, Odense, Denmark

[2] Molecular Diagnostics and Clinical Research Unit, Institute of Regional Health Research, University Hospital of Southern Denmark, Hospital Sønderjylland, Aabenraa, Denmark

[3] Section for Biostatistics and Evidence-Based Research, the Parker Institute, Bispebjerg and Frederiksberg Hospital, Copenhagen, Denmark

[4] Internal Medicine & Emergency Department Svendborg, Odense University Hospital, Svendborg, Denmark

[5] Research Unit of Medical Gastroenterology, Department of Clinical Research, University of Southern Denmark, Odense, Denmark

[6] Department of Gastroenterology, Landspitali University Hospital, Reykjavik, Iceland

[7] Department of Internal Medicine, Örebro University Hospital, Region Örebro län, Örebro, Sweden

[8] Department of Gastroenterology, Oslo University Hospital, Oslo, Norway

[9] Department of Internal Medicine–Gastroenterology, University Hospital of Southern Denmark, Lillebaelt Hospital, Vejle, Denmark

[10] Gastrointestinal Diseases, Nordic Bioscience A/S, Herlev, Denmark

[11] Research Unit of Medical gastroenterology and hepatology, University Hospital of Southern Denmark, Hospital South West Jutland, Esbjerg, Denmark

[12] Jönköping Academy for Improvement in Health and Welfare, Jönköping University, Jönköping, Sweden

[13] Futurum-Academy for Healthcare, Region Jönköping County, Jönköping, Sweden



For peer review only

[14] School of Health and Medical Sciences, Örebro University, Örebro, Sweden

[15] Department of Biomedical and Clinical Sciences, Linköping University, Linköping, Sweden

[16] Institute of Regional Health Research, University of Southern Denmark, Odense, Denmark

[17] Institute of Molecular Medicine, University of Southern Denmark, Odense, Denmark

[18] OPEN - Open Patient data Explorative Network, University of Southern Denmark, Odense, Denmark

[19] Research Unit of Rheumatology, Department of Clinical Research, University of Southern Denmark, Odense University Hospital, Odense, Denmark

[20] Institute of Clinical Medicine, University of Oslo, Oslo, Norway

Acknowledgements

Acknowledgements are based on the ICMJE recommended criteria for authorship.[38]

Professor Björnsson ES, Professor Carlson M, Professor Heitmann BL, associate Professor Hov JR, CEO in Bio-Me Isaksen M, Professor Kruse TA, Professor Moum B, associate Professor Ophei R, MD, PhD Ricanek P, and senior coordinator and innovation advisor Wallin Å contributed to the design of the study. The Open Patient data Explorative Network (OPEN), Odense University Hospital, Region of Southern Denmark has facilitated storage and documentation of the biological material collected in the OPEN biobank framework. Thanks to research advisor, MPH Caroline Moos for English text editing. Thanks to the Danish Colitis-Crohn's Association, the Norwegian Mage-tarmforbundet, the Swedish Mag- och tarmförbundet, and the Icelandic patient representatives for valuable advice when planning and conducting the NORDTREAT study.



For peer review only - http://bmjopen.bmj.com/site/about/guidelines.xhtml

Authors' contributions (ORCID in parentheses)

Fejrskov A (0000-0002-1257-8630), Füchtbauer JD (0000-0002-9591-2963), Andersen V (0000-0002-0127-2863), Christensen R (0000-0002-6600-0631), and Kjeldsen J (0000-0001-8148-6572) wrote the first draft. Davíðsdóttir LG, Halfvarson J (0000-0003-0122-7234), Høivik ML (0000-0002-0104-465X), Mortensen JH (0000-0003-0326-7264), Rejler M (0000-0003-3286-2898), Repsilber D (0000-0002-7173-5579), Söderholm JD (0000-0002-3250-5367), Andersen V, Christensen R, and Kjeldsen J contributed to the conception and design of the study and are all members of the Executive Committee. Jensen MD (0009-0004-8432-1764), Nielsen LN, and Aalykke C are PIs and contributed to the inclusion of patients in the Region of Southern Denmark. All authors accepted the final submitted version.

Funding

The NORDTREAT project has received funding from NordForsk (30,000,000 SKR) via Vinnova (grant number 2019-01185 NORDTREAT), Rannis (NordForsk no. 90569 – grant no. 199782-0611), The Research Council of Norway (grant no. 2988039), and Innovation Fund Denmark (90569 NORDTREAT – grant number 8114-00026B). The Danish partners have allocated the funding (4,800,000 DKR) for remuneration of project staff, biobanking, and analyses of biological material. Furthermore, the University Hospital of Southern Denmark, Hospital Sønderjylland has funded 1,125,000 DKR for Ph.D. salary (Fejrskov A [grant number not applicable]), The Region of Southern Denmark (Fri og Strategisk Forskning) has funded 1,137,000 DKR (Fejrskov A [J.no.: 21/17578 and Efond: 1084] and Füchtbauer JD [grant number A1381]) for biobanking and analyses of biological material. Knud and Edith Eriksen Memorial Fund has funded 75,000 DKR for Ph.D. salary (Fejrskov A [case no. 62786-2022]). Odense University Hospital has funded 584,000 DKR for Ph.D. salary (Füchtbauer JD [grant number A5031). Section for Biostatistics and Evidence-Based Research, the Parker Institute, Bispebjerg and Frederiksberg Hospital (Christensen R) is supported by a core grant from the Oak Foundation (OCAY-13-309). None of the researchers participating in the project have any financial connection to the funding organisation, nor do they have a financial interest in the study.



For peer review only

Competing interests

All authors declare no conflict of interest.

The funding sponsors had no role in the study's design; collection, management, analysis, or interpretation of data; in writing of the protocol, or the decision to submit results for publication.

Patient consent

Written informed consent will be obtained from participants after receiving verbal and written project information and before participating in the study.

Provenance and peer review

Not commissioned; externally peer-reviewed.

Open Access

This is an Open Access article distributed in accordance with the Creative Commons Attribution Non Commercial (CC BY-NC 4.0) license (https://creativecommons.org/ licenses/by-nc/4.0/). This license permits others to share, redistribute, adapt, and build upon the work non-commercially, and license their derivative works on different terms, provided the original work is properly cited and the use is non-commercial.



For peer review only

For peer review only - http://bmjopen.bmj.com/site/about/guidelines.xhtml

# REFERENCES


1 Zhao M, Gönczi L, Lakatos PL, *et al.* The Burden of Inflammatory Bowel Disease in Europe in 2020. *J Crohns Colitis*. 2021;15:1573–87.

2 Alatab S, Sepanlou SG, Ikuta K, *et al.* The global, regional, and national burden of inflammatory bowel disease in 195 countries and territories, 1990–2017: a systematic analysis for the Global Burden of Disease Study 2017. *Lancet Gastroenterol Hepatol*. 2020; 5:17–30.

3 Ng SC, Shi HY, Hamidi N, *et al.* Worldwide incidence and prevalence of inflammatory bowel disease in the 21st century: a systematic review of population-based studies. *The Lancet*. 2017; 390:2769–78.

4 Kaplan GG, Windsor JW. The four epidemiological stages in the global evolution of inflammatory bowel disease. *Nat Rev Gastroenterol Hepatol*. 2021; 18:56–66. doi: 10.1038/s41575-020-00360-x

5 Maaser C, Sturm A, Vavricka SR, *et al.* ECCO-ESGAR Guideline for Diagnostic Assessment in IBD Part 1: Initial diagnosis, monitoring of known IBD, detection of complications. *J Crohns Colitis*. 2019; 13:144-164K.

6 Binder V, Both H, Hansen PK, *et al.* Incidence and prevalence of ulcerative colitis and Crohn's disease in the County of Copenhagen, 1962 to 1978. *Gastroenterology*. 1982; 83:563–8.

7 Lennard-Jones JE, Shivananda S. Clinical uniformity of inflammatory bowel disease a presentation and during the first year of disease in the north and south of Europe. EC-IBD Study Group. *Eur J Gastroenterol Hepatol*. 1997; 9:353–9.

8 Schultze JL, Rosenstiel P. Systems Medicine in Chronic Inflammatory Diseases. *Immunity*. 2018; 48:608–13.

9 Vicente AM, Ballensiefen W, Jönsson J-I. How personalised medicine will transform healthcare by 2030: the ICPerMed vision. *J Transl Med*. 2020; 18:180.

10 Vision Paper on Personalised Medicine Research & Implementation by 2030 - ICPerMed. https://www.icpermed.eu/en/activities-vision-paper.php (accessed 13 September 2023)

11 Thorlacius-Ussing G, Schnack Nielsen B, Andersen V, *et al.* Expression and Localization of miR-21 and miR-126 in Mucosal Tissue from Patients with Inflammatory Bowel Disease. *Inflamm Bowel Dis*. 2017; 23:739–52.




For peer review only

12 Zhou Y, Xu ZZ, He Y, *et al.* Gut Microbiota Offers Universal Biomarkers across Ethnicity in Inflammatory Bowel Disease Diagnosis and Infliximab Response Prediction. *mSystems*. 2018;3:e00188-17.

13 Mortensen JH, Manon-Jensen T, Jensen MD, *et al.* Ulcerative colitis, Crohn's disease, and irritable bowel syndrome have different profiles of extracellular matrix turnover, which also reflects disease activity in Crohn's disease. *PLoS ONE*. 2017; 12:e0185855.

14 Bennike T, Birkelund S, Stensballe A, *et al.* Biomarkers in inflammatory bowel diseases: Current status and proteomics identification strategies. *World J Gastroenterol WJG*. 2014; 20:3231–44.

15 van Rheenen PF, Van de Vijver E, Fidler V. Faecal calprotectin for screening of patients with suspected inflammatory bowel disease: diagnostic meta-analysis. *BMJ*. 2010;341:c3369.

16 Mortensen JH, Sinkeviciute D, Manon-Jensen T, *et al.* A Specific Calprotectin Neo-epitope [CPa9-HNE] in Serum from Inflammatory Bowel Disease Patients Is Associated with Neutrophil Activity and Endoscopic Severity. *J Crohns Colitis*. 2022; 16:1447–60.

17 Mortensen JH, Godskesen LE, Jensen MD, *et al.* Fragments of Citrullinated and MMP-degraded Vimentin and MMP-degraded Type III Collagen Are Novel Serological Biomarkers to Differentiate Crohn's Disease from Ulcerative Colitis. *J Crohns Colitis*. 2015; 9:863–72.

18 Schroeder KW, Tremaine WJ, Ilstrup DM. Coated Oral 5-Aminosalicylic Acid Therapy for Mildly to Moderately Active Ulcerative Colitis. *N Engl J Med*. 1987; 317:1625–9.

19 Daperno M, D'Haens G, Van Assche G, *et al.* Development and validation of a new, simplified endoscopic activity score for Crohn's disease: the SES-CD. *Gastrointest Endosc*. 2004; 60:505–12.

20 Silverberg MS, Satsangi J, Ahmad T, *et al.* Toward an Integrated Clinical, Molecular and Serological Classification of Inflammatory Bowel Disease: Report of a Working Party of the 2005 Montreal World Congress of Gastroenterology. *Can J Gastroenterol*. 2005; 19:5A-36A.

21 Feng Y-S, Kohlmann T, Janssen MF, *et al.* Psychometric properties of the EQ-5D-5L: a systematic review of the literature. *Qual Life Res*. 2021; 30:647–73.

22 Ware JE, Sherbourne CD. The MOS 36-Item Short-Form Health Survey (SF-36): I. Conceptual Framework and Item Selection. *Med Care*. 1992; 30:473–83.

23 Guyatt G, Mitchell A, Irvine EJ, *et al.* A new measure of health status for clinical trials in inflammatory bowel disease. *Gastroenterology*. 1989; 96:804–10.

24 Iwine EJ, Fagan BG, Wang' CJ. of a Quality of Life Index for Inflammatory Bowel Disease Change the Results? *Qual Life*.




For peer review only - http://bmjopen.bmj.com/site/about/guidelines.xhtml

25 Vestergaard C, Dahlerup JF, Bager P. Validation of the Danish version of Inflammatory Bowel Disease Self-assessment Scale. 2017.

26 Palsson OS, Whitehead WE, Van Tilburg MAL, *et al.* Development and Validation of the Rome IV Diagnostic Questionnaire for Adults. *Gastroenterology*. 2016; 150:1481–91.

27 Harvey RF, Bradshaw JM. A SIMPLE INDEX OF CROHN'S-DISEASE ACTIVITY. *The Lancet*. 1980; 315:514.

28 Walsh AJ, Bryant RV, Travis SPL. Current best practice for disease activity assessment in IBD. *Nat Rev Gastroenterol Hepatol*. 2016; 13:567–79.

29 The Danish Health Examination Survey 2007-2008 (DANHES 2007-2008). https://doi.org/10.1177/1403494810393557

30 Maaser C, Sturm A, Vavricka SR, *et al.* ECCO-ESGAR Guideline for Diagnostic Assessment in IBD Part 1: Initial diagnosis, monitoring of known IBD, detection of complications. *J Crohns Colitis*. 2019; 13:144-164K.

31 Turner D, Ricciuto A, Lewis A, *et al.* STRIDE-II: An Update on the Selecting Therapeutic Targets in Inflammatory Bowel Disease (STRIDE) Initiative of the International Organization for the Study of IBD (IOIBD): Determining Therapeutic Goals for Treat-to-Target strategies in IBD. *Gastroenterology*. 2021; 160:1570–83.

32 Brodersen JB, Knudsen T, Kjeldsen J, *et al.* Diagnostic accuracy of pan-enteric capsule endoscopy and magnetic resonance enterocolonography in suspected Crohn's disease. *United Eur Gastroenterol J*. 2022; 10:973–82.

33 Burisch J, Katsanos KH, Christodoulou DK, *et al.* Natural Disease Course of Ulcerative Colitis During the First Five Years of Follow-up in a European Population-based Inception Cohort—An Epi-IBD Study. *J Crohns Colitis*. 2019; 13:198–208.

34 Burisch J, Andersen V, Čuković-Čavka S, *et al.* DOP019 Immunomodulators reduce the risk of surgery and hospitalisation in Crohn's disease in a prospective European population-based inception cohort: the Epi-IBD cohort. *J Crohns Colitis*. 2018;12:S042–3.

35 Bossuyt PM, Reitsma JB, Bruns DE, *et al.* STARD 2015: an updated list of essential items for reporting diagnostic accuracy studies. *BMJ*. 2015;351:h5527.

36 Collins GS, Reitsma JB, Altman DG, *et al.* Transparent reporting of a multivariable prediction model for individual prognosis or diagnosis (TRIPOD): the TRIPOD statement. BMJ. 2015;350:g7594.

37 von Elm E, Altman DG, Egger M, *et al.* Strengthening the reporting of observational studies in epidemiology (STROBE) statement: guidelines for reporting observational studies. BMJ. 2007; 335:806–8.



For peer review only - http://bmjopen.bmj.com/site/about/guidelines.xhtml

38 icmje-recommendations.pdf.https://www.icmje.org/icmje-recommendations.pdf (accessed 4 January 2023)

For peer review only



For peer review only - http://bmjopen.bmj.com/site/about/guidelines.xhtml

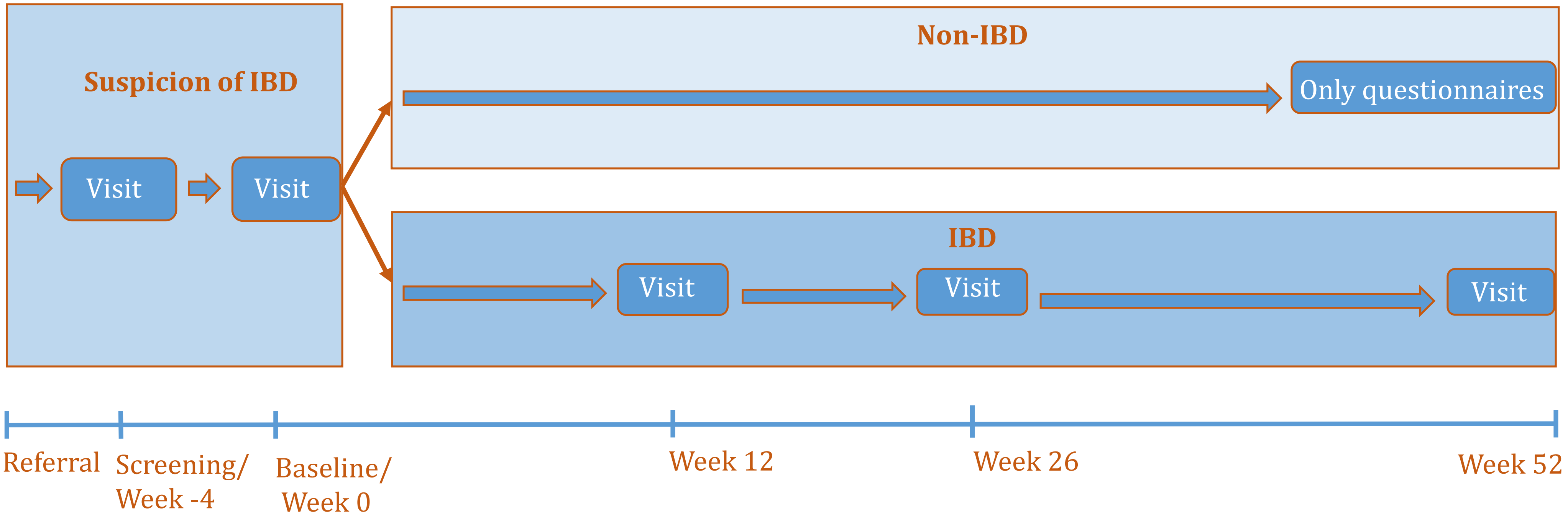
Suspicion of IBD
Visit
Visit
Non-IBD
Only questionnaires
IBD
Visit
Visit
Visit
Referral
Screening/
Week -4
Baseline/
Week 0
Week 12
Week 26
Week 52

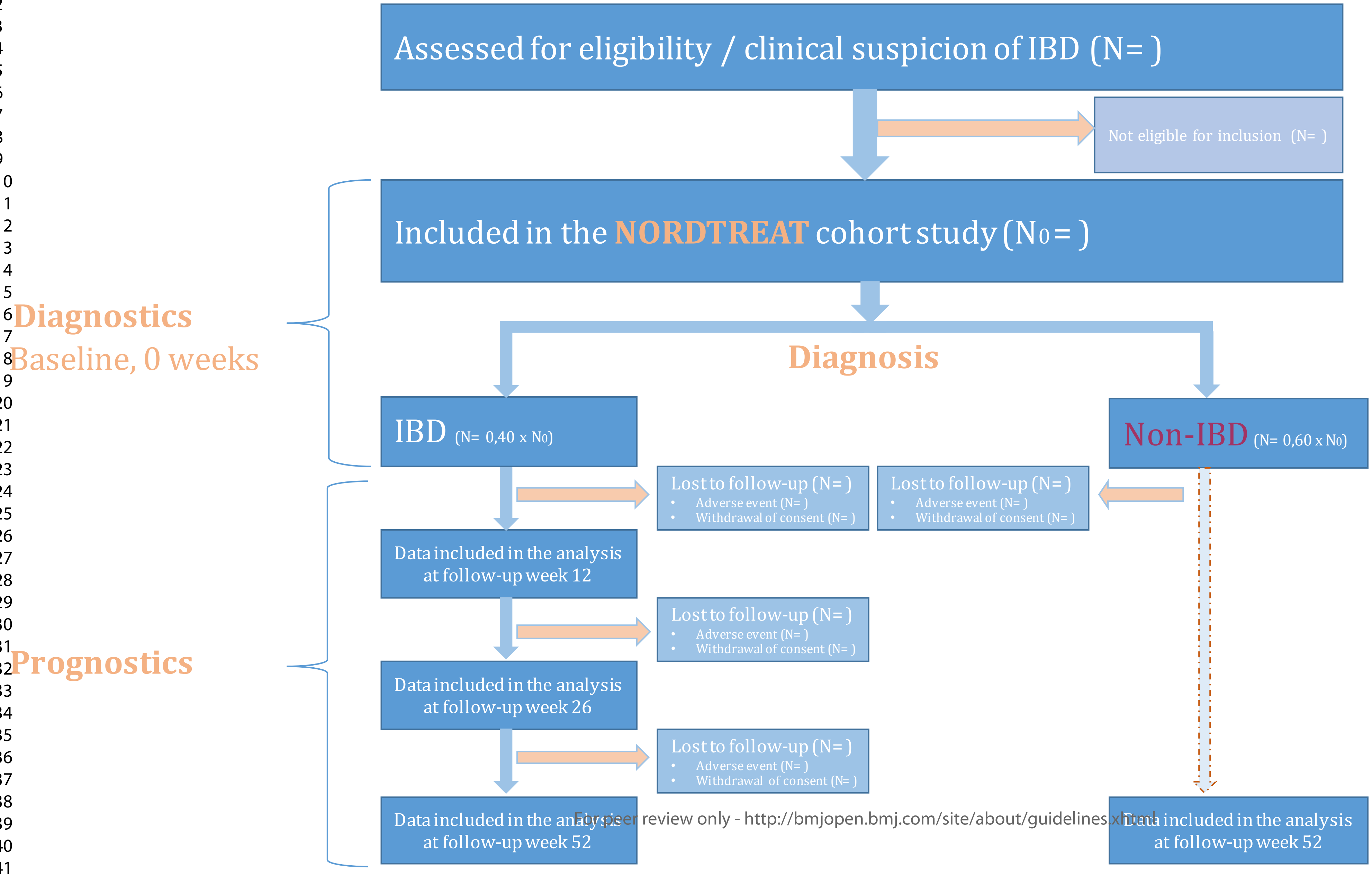
Assessed for eligibility / clinical suspicion of IBD (N= )
Not eligible for inclusion (N= )
Included in the NORDTREAT cohort study (N0 = )
Diagnostics
Baseline, 0 weeks
Diagnosis
IBD (N= 0,40 x N0)
Non-IBD (N= 0,60 x N0)
Lost to follow-up (N= )
Adverse event (N= )
Withdrawal of consent (N= )
Lost to follow-up (N= )
Adverse event (N= )
Withdrawal of consent (N= )
Data included in the analysis at follow-up week 12
Lost to follow-up (N= )
Adverse event (N= )
Withdrawal of consent (N= )
Prognostics
Data included in the analysis at follow-up week 26
Lost to follow-up (N= )
Adverse event (N= )
Withdrawal of consent (N= )
Data included in the analysis at follow-up week 52
Data included in the analysis at follow-up week 52

**Appendix 1:** Inclusion sites in the NORDTREAT Cohort study

| Country | Hospital |
|---|---|
| DK | Odense University Hospital |
| DK | OUH Svendborg Hospital |
| DK | SLB Vejle Hospital |
| DK | SVS Esbjerg Hospital |
| IS | Landspitali Reykjavik |
| NO | Oslo University Hospital |
| NO | Vestfold Hospital |
| NO | Drammen Hospital |
| NO | Skien Hospital |
| SE | Örebro University Hospital |
| SE | Högland Hospital, Eksjö |

For peer review only - http://bmjopen.bmj.com/site/about/guidelines.xhtml

NORDTREAT

SOP name: General blood sampling procedure Version V1.1Approval Date: 28.03.2022

## General blood sampling procedures

**Concerning:**

| | |
|---|---|
| The NORDTREAT treatment strategy trial (WP2) | x |
| The NORDTREAT prospective cohort study (WP3) | x |

**Blood sampling procedure for Screening/(Baseline) and week 52**

**This procedure is for the following time points**

| Visit | Screening/ (Baseline) | 12 weeks | 26 weeks | 52 weeks |
|---|---|---|---|---|
| Collected | x | x | x | x |

- Blood should not be drawn when the clensing procedure for colonoscopy is ongoing or on the day of colonoscopy (due to possible effects of bowel cleansing and colonoscopy procedures)
- Use a Vacuette Butterfly 21g 19cm (green). This sterile disposable cannula with a hose facilitates sampling and prevents backflow of liquid from the PAXgene® tubes.

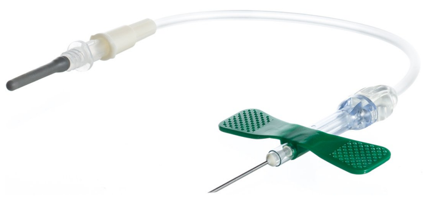

- Because of the hose, 1-2 ml of blood must be drawn in to an empty tube («throw away tube» ) before the first tube for biobanking is drawn.
- Then draw the blood in the following order:
  - 1. Serum tubes (for details see SOPs for Serum WP2 and Serum WP3)
  - 2. EDTA tubes (for details see SOPs for EDTA plasma WP3 and Whole blood WP3)
  - 3. PAXgene® tube (for details see SOP for PAXgene® WP3)(only at Screening/(Baseline) and week 52)

Responsible: Marte Lie Høivik

For peer review only - http://bmjopen.bmj.com/site/about/guidelines.xhtml

NORDTREAT

SOP name: Serum WP3Version V1.0Approval Date: 17.01.2022

# Serum

Concerning:

| | |
|---|---|
| The NORDTREAT treatment strategy trial (WP2) | |
| The NORDTREAT prospective cohort study (WP3) | x |

This material is collected at the following time points

| Visit | Screening/(Baseline) | 12 weeks | 26 weeks | 52 weeks |
|---|---|---|---|---|
| Collected | x | x | x | x |

### Material needed

- Two Serum tubes 10 ml Vacuette (draws 9 ml blood) (455092) use containers with a red cap
- 12 tubes for biobanking (e.g. 2 ml microtubes Sarstedt 72608 )

### Labelling

Containers are labeled as XX-YY-ZZZ-SO-V[b] using centrally provided labels from national coordinator.

[b]XX=country code, YY=centre code, ZZZ=subject code, SO=serum observention and V=visit code (where -2=screening, 0=baseline, 12=week 12, 26=week 26, and 52=week 52))

### Procedure

The serum must **be stored at -80°C no later than two hours after the blood is drawn**.

a) Leave the tube for 60 minutes at room temperature for clot formation
b) Remove the clot by centrifugation at 2.000 x G for 10 minutes at room temperature.
c) The serum is pipetted to the following aliquots: (Of note, sufficient volume of serum should be left in the container above the pellet)
   - 12 aliquots of 500µL

   Each aliquot is labeled as XX-YY-ZZZ-SI-V[b], using centrally provided labels from the national coordinator.
d) Storage at -80°C

Responsible: Marte Lie Høivik

For peer review only - http://bmjopen.bmj.com/site/about/guidelines.xhtml

NORDTREAT

SOP name: Serum WP3Version V1.0Approval Date: 17.01.2022

For peer review only

Responsible: Marte Lie Høivik

NORDTREAT

## SOP name: EDTA whole blood WP3Version V1.0Approval Date: 17.01.2022

## Whole blood

**Concerning:**

| | |
|---|---|
| The NORDTREAT treatment strategy trial (WP2) | |
| The NORDTREAT prospective cohort study (WP3) | x |

**This material is collected at the following time points**

| Visit | Baseline/inclusion | 12 weeks | 26 weeks | 52 weeks |
|---|---|---|---|---|
| Collected | x | | | |

## Material needed

- One 4 ml K2EDTA Vacuette (454209 4ml)
- Two 3,5 ml Nunc tubes

## Labelling

Each tube is labeled as XX-YY-ZZZ-WH-V[b], using centrally provided labels from the national coordinator

[b]XX=country code, YY=centre code, ZZZ=subject code, WH=whole blood and V=visit code where 0=baseline

## Procedure sampling and handling

a) Gently mix 8-10 times immediately after sampling.
b) No centrifugation. Aliqout approximately equal volumes into two Nunc tubes.
c) The material must be stored at -80°C no later than two hours after the blood is drawn.

Responsible: Marte Lie Høivik

For peer review only

NORDTREAT

## SOP name: EDTA plasma WP3Version V1.0Approval Date:17.01.2022

## EDTA plasma

**Concerning:**

| | |
|---|---|
| The NORDTREAT treatment strategy trial (WP2) | |
| The NORDTREAT prospective cohort study (WP3) | x |

**This material is collected at the following timepoints**

| Visit | Screening/(Baseline) | 12 weeks | 26 weeks | 52 weeks |
|---|---|---|---|---|
| Collected | x | x | x | x |

## Material needed

- One 10 ml K2EDTA Vacuette collection tubes (455045) (violet cap)
- 9 tubes for biobanking (2 ml microtubes e.g. Sarstedt 72608 )

## Labelling

Each tube is labeled as XX-YY-ZZZ-P- V[b], using centrally provided labels from the national coordinator.

[b]XX =country code, YY=centre code, ZZZ=subject code, P=plasma and V=visit code (where -2= screening, 0=baseline, 12=week 12, 26=week 26, and 52=week 52)

## Procedure

a) Gently mix 8-10 times immediately after sampling.
b) Centrifuge immediately at 2.000 G for 10 minutes in a room tempered centrifuge.
c) Plasma is pipetted to 9 aliquots of (at least) 500 µL, at least 1 ml of plasma should be left in the container above the pellet.
d) The material must be stored at -80°C no later than two hours after the blood is drawn.

Responsible: Marte Lie Høivik

For peer review only - http://bmjopen.bmj.com/site/about/guidelines.xhtml

For peer review only

NORDTREAT

## SOP name: PAXgene® WP3Version V1.1Approval Date: 28.03.2022

## PAXgene®

**Concerning:**

| | |
|---|---|
| The NORDTREAT treatment strategy trial (WP2) | |
| The NORDTREAT prospective cohort study (WP3) | x |

**The material is collected at following time points:**

| Visit | Screening/(Baseline) | 12 weeks | 26 weeks | 52 weeks |
|---|---|---|---|---|
| Collected | X | | | X |

## Material needed

- One PAXgene® (RNase free) 2.5 ml tube

## Labelling

Each tube is labeled as XX-YY-ZZZ-Px-V[b], using centrally provided labels from the national coordinator.

[b]XX=country code, YY=centre code, ZZZ=subject code, Px= PAXgene and V=visit code (where -2= screening, 0=baseline and 52=week 52)

## Procedure for sampling and handling of tubes

The PAXgene® (RNase free) containers are stored at room temperature (18-25°C) before use

a) PAXgene® is to be drawn as the last container (see SOP General blood sampling procedure)
b) The tube is held vertically below the patient's arm during drawing of blood
c) Use at least 10 seconds to draw the blood and to ensure that blood flow has stopped before removing the tube.
d) Gently turn 8-10 times immediately after sampling
e) Store the PAXgene® tubes upright at room temperature (18-25°C) for 2 hours
f) Freeze in an upright position at -20°C for a minimum of 24 and a maximum of 72 hours
g) Move to storage at -80°C

Responsible: Marte Lie Høivik

For peer review only - http://bmjopen.bmj.com/site/about/guidelines.xhtml

NORDTREAT

SOP name: Feces WP3 Version V1.0Approval Date: 17.01.2022

## Feces

Concerning:

| | |
|---|---|
| The NORDTREAT treatment strategy trial (WP2) | |
| The NORDTREAT prospective cohort study (WP3) | x |

This material is collected at the following time points

| Visit | Baseline | 12 weeks | 26 weeks | 52 weeks |
|---|---|---|---|---|
| Collected | x | x | x | x |

## Material needed

- Stool DNA Stabilizer collection tubes Stool Collection Tubes with DNA Stabilizer Art nr: 1038111300 ( https://www.nordicbiolabs.se/nordic-biolabs-ab/molekylarbiologi/extraction-purification/genomiskt-dna/feces-saliv/stool-dna-stabilizer-collection-tubes )
- Dry collection tube for stool samples
- Transport tubes
- Transport envelope

## General information.

- Fecal samples for pathogens and Clostridium difficile are collected according to local lab routine
- At inclusion and at 52 weeks, the **fecal samples should be taken from the first stool the day before bowel cleansing is initiated (as part as the preparations for the colonoscopy). At the other visits (12 weeks, 26 weeks and extra visits) the samples should be taken on the day before or the day og the visit, if possible.**
- If the fecal sample has not obtained before colonoscopy, the patient should be instructed to provide it 3-7 days after colonoscopy.
- Detailed written instructions for sampling are included in the patient folder and should be reviewed with the patient.

## Labeling

The collection tubes are labeled as XX-YY-ZZZ-FS-V[a] or XX-YY-ZZZ-FD-V[a] using centrally provided labels from the national coordinator.

[a]XX=country code, YY=centre code, ZZZ=subject code, FS=feces stabilizer and FD= feces dry and V=visit code (where 0=baseline, 12=week 12, 26=week 26, and 52=week 52)

Responsible: Marte Lie Høivik

For peer review only

NORDTREAT

SOP name: Feces WP3 Version V1.0Approval Date: 17.01.2022

### Procedure for handing out fecal sample material

- At the screening visit, hand out the material for the baseline sample
- Provide the patient with information on how and when to collect fecal samples, **and how to record the date of collection**.
- After the Baseline/inclusion visit, hand out the remaining fecal sample kits in advance of each visit
- The fecal samples are brought by the patient to the hospital at each visit/endoscopy. If the patient is not scheduled for a visit, inform the patient about how to send the sample to the biobank (this according to national procedure)

### Content in Fecal sample kit per visit

- Detailed written information for sampling
- Sampling material
- One tube with stabiliser (marked "shake well") and one dry tube.
- The collection tubes are labeled as XX-YY-ZZZ-FS-V[a] or XX-YY-ZZZ-FD-V[a] using centrally provided labels from the national coordinator

(If sendt by post: Two transport containers and one transport envelope pre-payed and pre-addressed (according to national procedure))

### Sampling is described in the written information

In breaf

- o In tubes with DNA preserving additives are one spoonful of feces (there is a spoon in the tube lid) is put in the tube. Lock the lid and shake well. Write the collection date on the label
- o In dry tubes are The container filled half with feces. There is a spoon in the lid. Close the lid. No need for shaking. Write the collection date on the label

Put the two tubes in the transport tubes, and bring everything to the hospital visit or put everything in in the pre-addressed envelope and mail at first convenience.

### Biobanking

- Record the date of fecal collection and the date of when the biobank received the sample
- The dry tubes are frozen at -80C in the collection tube
- The tubes with DNA preserving additives are frozen at -80C in the collection tube

Responsible: Marte Lie Høivik

For peer review only - http://bmjopen.bmj.com/site/about/guidelines.xhtml

NORDTREAT

SOP name: Feces_RNALater_WP3_Optional_Version V1.0 Approval Date: 28.03.2022

## Feces

Concerning:

| | |
|---|---|
| The NORDTREAT treatment strategy trial (WP2) | |
| The NORDTREAT prospective cohort study (WP3 – Danish sites) | x |

The material is collected at the following time points

| Visit | Baseline | 12 weeks | 26 weeks | 52 weeks |
|---|---|---|---|---|
| Collected | x | x* | x* | x* |

*** Only IBD pt. (non-IBD will only hand in samples at baseline)**

## Material needed

- Stool collection tube containing RNALater solution (ThermoFisher: https://www.thermofisher.com/order/catalog/product/AM7021)
- Transport tube
- Transport envelope

## General information.

- Fecal samples for pathogens and Clostridium difficile are collected according to local lab routine
- At inclusion and at 52 weeks, the **fecal sample should be taken from the first stool the day before bowel cleansing is initiated (as part of the preparations for the colonoscopy). At the other visits (12 weeks, 26 weeks and extra visits) the sample should be taken on the day before or the day of the visit, if possible.**
- If the fecal sample has not been obtained before colonoscopy, the patient should be instructed to provide it 3-7 days after colonoscopy.
- Detailed written instructions for sampling are included in the patient folder " Prøvetagning i eget hjem – Afføringsprøve" and should be reviewed with the patient.

## Labeling

The collection tubes are labeled as XX-YY-ZZZ-FR-V[a] using centrally provided labels from the national coordinator.

[a]XX=country code, YY=centre code, ZZZ=subject code, FR=feces RNA stabilizer and V=visit code (where 0=baseline, 12=week 12, 26=week 26, and 52=week 52)

Responsible: Anja Fejrskov Severino

For peer review only - http://bmjopen.bmj.com/site/about/guidelines.xhtml

NORDTREAT

## SOP name: Feces_RNALater_WP3_Optional_Version V1.0 Approval Date: 28.03.2022

### Procedure for handing out fecal sample material

- At the screening visit, hand out the material for the baseline sample
- Provide the patient with information on how and when to collect the fecal samples, **and how to record the date of collection**.
- After the Baseline/inclusion visit, hand out the remaining fecal sample kits in advance of each visit
- The fecal samples are brought by the patient to the hospital at each visit/endoscopy. If the patient is not scheduled for a visit, inform the patient how to send the sample to the biobank

### Content in Fecal sample kit per visit

- Detailed written information for sampling: " Prøvetagning i eget hjem – Afføringsprøve"
- One stool collection tube with 5 ml RNALater stabiliser
- The collection tube is labeled as XX-YY-ZZZ-FR-V[a] using centrally provided labels from the national coordinator
- Sample requisition paper with study ID and field for sample collection date.

(If sent by post: One transport container and one transport envelope pre-payed and pre-addressed)

### Sampling is described in the written information

In brief

- In the tube with RNALater preserving additives four spoonful of feces from different sites are put (there is a spoon in the lid of the tube). Lock the lid and shake well. Write the collection date on the sample requisition paper in the appropriate field.
- Put the stool collection tube in the transport tube, and bring everything to the hospital visit or put everything in in the pre-addressed envelope and mail at first convenience.

### Biobanking

- Record the date of fecal collection and the date of when the biobank received the sample
- The fecal sample is aliquoted in 3 micro tubes (2 ml) and frozen at -80°C

Responsible: Anja Fejrskov Severino



For peer review only

NORDTREAT

## SOP name: Gut mucosal biopsies WP3 Version V1.1Approval Date: 28.03.2022

# Gut mucosal biopsies in Allprotect®

**Concerning:**

| | |
|---|---|
| The NORDTREAT prospective cohort study (WP3) | X (study biopsies) |

**This material is collected at the following time points**

| Visit | Baseline | 12 weeks | 26 weeks | 52 weeks |
|---|---|---|---|---|
| Collected | x | | | X |

## Material needed

8 psc 0,5 ml containers (e.g. Sarstedt # 72.694.007) filled with 150 µl Allprotect®

## Labelling

Biopsies are labeled as XX-YY-ZZZ-B(I-VIII)-V[b], using centrally provided labels from the national coordinator.

[b]XX=country code, YY=centre code, ZZZ=subject code, B=biopsy number using Roman numbers, i.e. I-VIII and V=visit code where 0=baseline and 52=week 52. For further details see table below!

## Procedure for obtaining biopsies

**Where to obtain biopsies**

- Biopsies should be obtained from both inflamed and non-inflamed segments according to the table below
- For biopsies from inflamed mucosa, the most inflamed area in the segment should be biopsied

**Handling of the biopsies**

a) Standard jumbo biopsy forceps are used and one biopsy should be taken for each tube.
b) The biopsies must immediately be lowered into the liquid of Allprotect® and the biopsy must be totally covered by Allprotect® to protect DNA, RNA and proteins.
c) Incubated for a minimum of 24 and maximum of 72 hours in fridge at 2-8 °C.
d) Stored at -80 °C.

Responsible: Marte Lie Høivik

For peer review only - http://bmjopen.bmj.com/site/about/guidelines.xhtml

NORDTREAT

## SOP name: Gut mucosal biopsies WP3 Version V1.1Approval Date: 28.03.2022

The table is used during endoscopy when taking biopsies and recording. For each segment of biopsy, make a notification in the table and upload the information into Viedoc. In UC and Crohns colitis with no non-inflamed segments of the colon, biopsies III and IV shall not be taken.

1. Tick correct diagnosis and if biopsies are taken in the table.
2. Mark site were biopsies are taken, and inflammation status on the figure.
3. Label biopsies according to "biopsy no" in the table using preprintet labels
4. Record information on site and inflammation status of biopsy 1 -8 in Viedoc.

| 1)N Suggested diagnose at endoscopy (tick) | | | 2) Biopsy no | | | 2) | | | 2) Biopsy no | | | |
|---|---|---|---|---|---|---|---|---|---|---|---|---|
| | | | I | II | | III | IV | | V | VI | VII | VIII |
| | UC | Term ileum n=2 | | | 10cm prox of inflammation n=2 | | | Rectum inflamed segment n=4 | | | | |
| | Crohns colitis | Term ileum n=2 | | | 10 cm prox of inflammation n=2 | | | Most distal inflamed segment n=4 | | | | |
| | Ileocolonic | Term ileum n=2 (inflamed part) | | | 10 cm prox of inflammation n=2 | | | Most distal inflamed segment n=4 | | | | |
| | Ileal Crohn | Term ileum n=2 (inflamed part) | | | Left colon n=2 | | | Rectum n=4 | | | | |
| | Normal endoscopy | Term ileum n=2 | | | Left colon n=2 | | | Rectum n=4 | | | | |

If a biopsies are taken from this segment, mark the box with:

I= inflamed
N= non-inflamed
P= proximal

If video is recorded in this segment, tick the circle

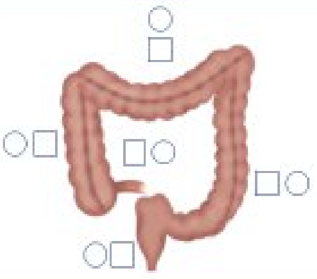

NORDTREAT

Responsible: Marte Lie Høivik

NORDTREAT

## SOP name: Snap freeze of biopsies in liquid nitrogen or FlashFreeze
## Version V1.2 Approval Date: 03.11.2022

## Snap freeze of biopsies in liquid nitrogen or FlashFreeze

Concerning:

| | |
|---|---|
| The NORDTREAT treatment strategy trial | |
| The NORDTREAT prospective cohort study | X (study biopsies) |

The material is collected at the following time points

| Visit | Baseline | 12 weeks | 26 weeks | 52 weeks |
|---|---|---|---|---|
| Collected | x | | | x |

This SOP describes the processing of tissue for snap freezing with liquid nitrogen or FlashFreeze.

## Material needed

Freezing in liquid nitrogen:

- Styrofoam container with dry ice
- Isolated transport container (0,5-1L) suitable for liquid nitrogen, filled approx. 2/3
- Sterile needle
- Forceps
- 6 psc. 0,5 ml micro tubes (e.g. Sarstedt # 72.694.007)
- Cryocompatible labels/marker

Additional material if freezing in FlashFreeze:

- FlashFreeze apparatus: https://www.milestonemedsrl.com/product/flashfreeze/
- Product instructions for the apparatus
- 99,9% pure ethanol

Personal Protective Equipment:

Safety glasses or face shield
Disposable latex or nitrile gloves
Gloves for handling liquid nitrogen
Clean laboratory coat

Responsible: David Füchtbauer

For peer review only - http://bmjopen.bmj.com/site/about/guidelines.xhtml

NORDTREAT

## SOP name: Snap freeze of biopsies in liquid nitrogen or FlashFreeze

## Version V1.2 Approval Date: 03.11.2022

## Labelling

Biopsies are labeled as XX-YY-ZZZ-B(IX-XIIII)-V[b], using centrally provided labels from the national coordinator.

[b]XX= country code, YY= centre code, ZZZ= subject code, B= biopsy number using Roman numbers, i.e. IX-XIIII and V= visit code where 0= baseline and 52= week 52. For further details see table below!

## Procedure

### Liquid nitrogen:

a) Label the micro tubes with patient ID, site and visit number (Labels are pre-printed)
b) Place one biopsy in each micro tube directly after sampling - use a sterile needle to gently nudge the tissue into the tube.
c) Place without delay the micro tube in liquid nitrogen, for at least 60 sec.
d) After initial freeze, keep the cryovials on dry ice, until long-term storage at -80°C.

### FlashFreeze:

If a FlashFreeze (-80°C ethanol) is used instead of liquid nitrogen, the same procedure is used, but following must be noted:

a) Set FlashFreeze apparatus to start 2 hours prior to the endoscopy. Check that the operating temperature (-76°C to -84°C) has been reached at start.
b) The vials must be in -80°C ethanol at least 120 sec. before they are moved to dry ice. (for transportation to long-term storage at -80°C)
c) Clean and defrost the apparatus as described in the product guide.

The table is used during endoscopy when taking biopsies and recording. For each segment of biopsy, make a notification in the table and upload the information into Viedoc. In UC and Crohns colitis with no non-inflamed segments of the colon, biopsies III and IV shall not be taken.

NORDTREAT | Patient ID: DK - 01 - ____ | Visit: baseline ☐ w52 ☐ | ODENSE

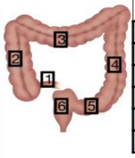


| 2) | Location | Video 15-30s No ID | I= inflamed N= non-inflamed P= proximal | Rutine biopsy | Study biopsy | ▲ Mayo score | SES-CD ◆ S | U | A | P |
|---|---|---|---|---|---|---|---|---|---|---|
| 1 | Terminal ileum | | | | | | | | | |
| 2 | Ascendens | | | | | | | | | |
| 3 | Transversum | | | | | | | | | |
| 4 | Descendens | | | | | | | | | |
| 5 | Sigmoideum | | | | | | | | | |
| 6 | Rectum | | | | | | | | | |

| 1) Suggested diagnose at endoscopy (tick) | | Biopsy no (tick if biopsies are taken) | | | Biopsy no. (tick if biopsies are taken) | | | Biopsy no (tick if biopsies are taken) | | |
|---|---|---|---|---|---|---|---|---|---|---|
| | Biopsy number | | 1+2 | 9+10 | | 3+4 | 11+12 | | 5-8 | 13+14 |
| | UC ▲ | Term ileum | | | 10cm prox of inflammation | | | Rectum inflamed segment | | |
| | Crohns colitis (▲) ◆ | Term ileum | | | 10 cm prox of inflammation | | | Most distal inflamed segment | | |
| | Ileocolonic (▲) ◆ | Term ileum (inflamed part) | | | 10 cm prox of inflammation | | | Most distal inflamed segment | | |
| | Ileal Crohn ◆ | Term ileum (inflamed part) | | | Left colon | | | Rectum | | |
| | Normal endoscopy | Term ileum | | | Left colon | | | Rectum | | |

1) Tick correct diagnosis and orientate where study biopsies are to be taken in blue table.
2) For every location mark video, inflammation status, rutine biopsy, study biopsy, Mayo score and SES-CD where applicable.
3) Biopsies must be labeled according to this chart with preprinted labels
4) Record information on site and inflammation status of biopsy 1 - 8 In Viedoc.

**Biopsy 1-8 in All-protect**

**Biopsy 9-14 fresh frozen**

◆ SES-CD

| | | |
|---|---|---|
| Size of ulcers (diameter) (S) | Absent | 0 |
| | Aphthous ulcers, 0.1–0.5 cm | 1 |
| | Large ulcers, 0.5–2 cm | 2 |
| | Very large ulcers, >2 cm | 3 |
| Ulcerated surface (U) | None | 0 |
| | <10% of the segment | 1 |
| | 10–30% of the segment | 2 |
| | >30% of the segment | 3 |
| Affected surface (A) | None | 0 |
| | <50% of the segment | 1 |
| | 50–75% of the segment | 2 |
| | >75% of the segment | 3 |
| Presence of narrowings (P) | None | 0 |
| | Single, passable by scope | 1 |
| | Multiple, passable by scope | 2 |
| | Not passable, frank stenosis | 3 |

▲ MAYO SCORE

An endoscopic scoring system for ulcerative colitis

| | |
|---|---|
| Score 0 | Normal or inactive disease |
| Score 1 | Mild disease (erythema, decreased vascular pattern, mild friability) |
| Score 2 | Moderate disease (marked erythema, absent vascular pattern, friability, erosions) |
| Score 3 | Severe disease (spontaneous bleeding, ulceration) |

Responsible: David Füchtbauer

For peer review only

NORDTREAT

## SOP name: Urine WP3Version V1.1Approval Date: 28.3.2022

# Urine

**Concerning:**

| | |
|---|---|
| The NORDTREAT treatment strategy trial (WP2) | |
| The NORDTREAT prospective cohort study (WP3) | x |

**This material is collected at the following time points**

| Visit | Baseline | 12 weeks | 26 weeks | 52 weeks |
|---|---|---|---|---|
| Collected | x | | | x |

## Material needed

- One container suitable for collection of spot urine.
- Two containers for aliquots á 4 ml (e.g. Nunc Cryotubes 4,5ml)

## Labeling

Each urine aliquot is labeled as XX-YY-ZZZ-U-V[b], using centrally provided labels from the national coordinator.

[b]XX=country code, YY=centre code, ZZZ=subject code, U=urine and V=visit code (where 0=baseline and 52=week 52)

## Procedure for urine sampling and handling

a) Spot urine – taken when the blood samples are collected.
b) The urine is pipetted to 2 aliquots of 4 ml, frozen and stored at -80 °C the same day as collection
c) If aliquotation in not performed immediately, the urine should be stored in fridge locally within 2 hours after collection until aliquotation can be performed

Responsible: Marte Lie Høivik

For peer review only - http://bmjopen.bmj.com/site/about/guidelines.xhtml

NORDTREAT

SOP name: Hair Version V1.2 Approval Date: 28.03.2022

## Hair

Concerning:

| | |
|---|---|
| The NORDTREAT treatment strategy trial | |
| The NORDTREAT prospective cohort study | x |

This material is collected at the following time points

| Visit | Baseline | 12 weeks | 26 weeks | 52 weeks |
|---|---|---|---|---|
| Collected | x | | | x |

## Material

- Scissors
- Comb
- Clamp to keep up buckle
- Clip or Hair elastic
- Aluminium foil
- Marker that can write on aluminium foil
- Tape
- Re-sealable plastic bag with ID number / name

## Procedure

- Prepare a re-sealable plastic bag containing a piece of alu foil, a hair elastic or a clip
- Put hair up with the buckle
- Take a thin tuft of hair using the tip of a comb or the tip of a clamp
- Put the clip or the hair elastic around the hair ½-1 cm from the scalp (it is important to know the direction of the hair growth)
- Cut hair as close to the scalp as possible
- Keep the clip/hair elastic on and put the hair in alu foil
- Use tape to fix the clip/hair elastic to the alu foil
- Mark with a line on the alu foil, which part of the hair was closest to the scalp
- Wrap the hair in alu foil and put it in a re-sealable bag
- Remember ID number on the bag

Responsible: Anja Fejrskov Severino

For peer review only

NORDTREAT

SOP name: Hair Version V1.2 Approval Date: 28.03.2022

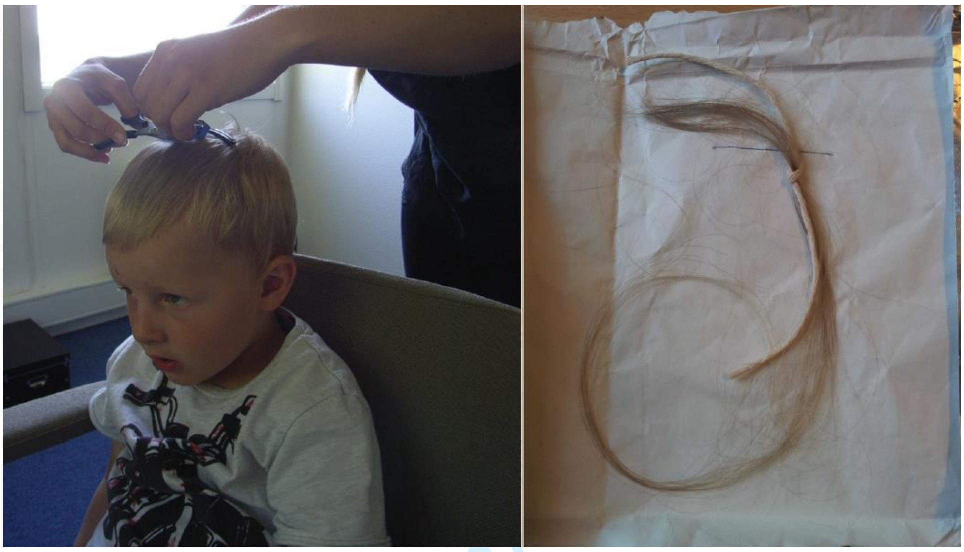

## Storage of hair samples

The hair samples will be stored at room temperature, with the unique participants ID placed on the bag.

Responsible: Anja Fejrskov Severino

For peer review only - http://bmjopen.bmj.com/site/about/guidelines.xhtml

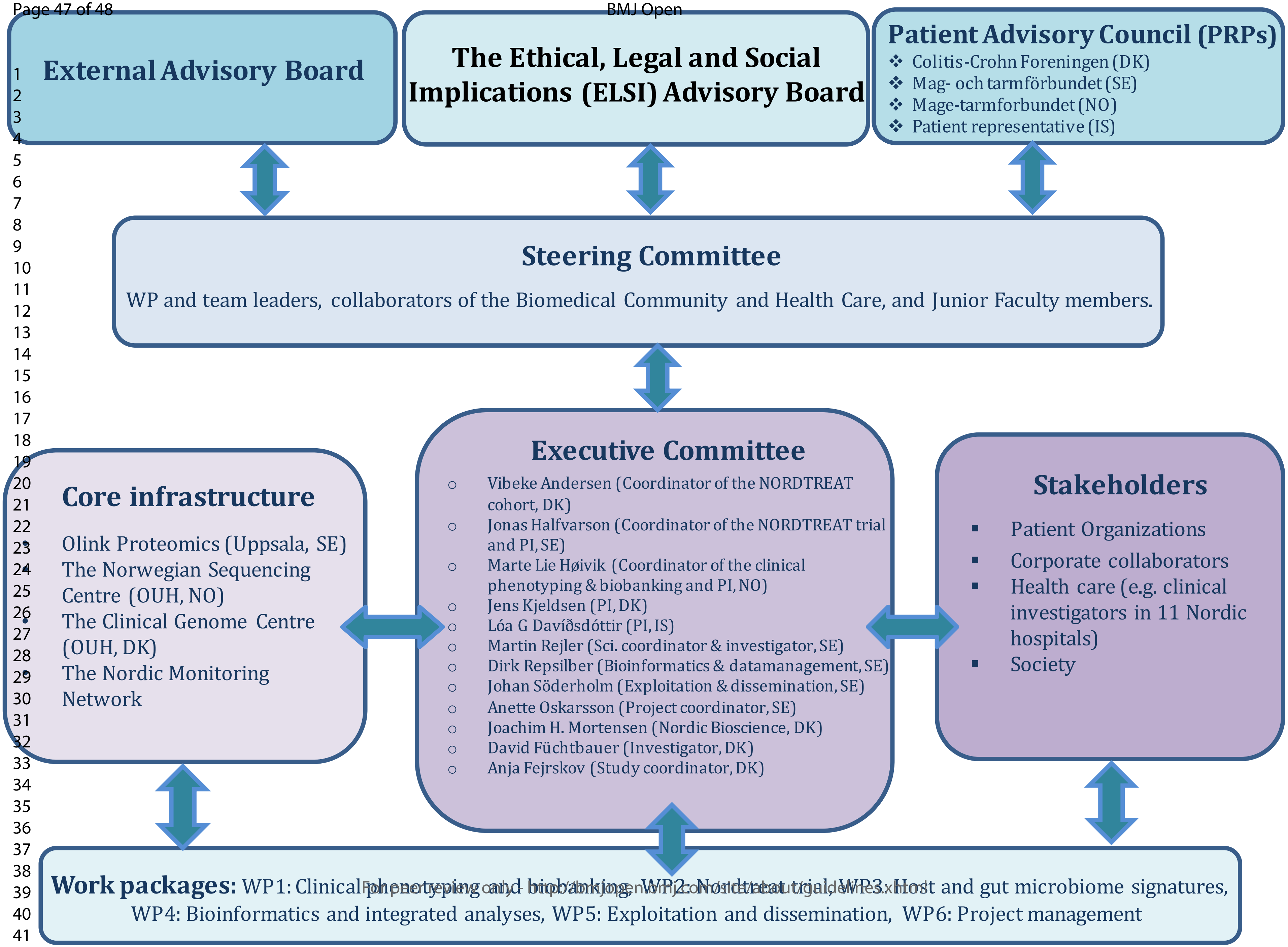


For peer review only - http://bmjopen.bmj.com/site/about/guidelines.xhtml

**STROBE 2007 (v4) Statement—Checklist of items that should be included in reports of *cohort studies***

| Section/Topic | Item # | Recommendation | Reported on page # |
|---|---|---|---|
| **Title and abstract** | 1 | (*a*) Indicate the study's design with a commonly used term in the title or the abstract | 1 |
| | | (*b*) Provide in the abstract an informative and balanced summary of what was done and what was found | 2 |
| **Introduction** | | | |
| Background/rationale | 2 | Explain the scientific background and rationale for the investigation being reported | 3-4 |
| Objectives | 3 | State specific objectives, including any prespecified hypotheses | 4-5 |
| **Methods** | | | |
| Study design | 4 | Present key elements of study design early in the paper | 5-6 |
| Setting | 5 | Describe the setting, locations, and relevant dates, including periods of recruitment, exposure, follow-up, and data collection | 6 |
| Participants | 6 | (*a*) Give the eligibility criteria, and the sources and methods of selection of participants. Describe methods of follow-up | 6 and 10 |
| | | (*b*) For matched studies, give matching criteria and number of exposed and unexposed | na |
| Variables | 7 | Clearly define all outcomes, exposures, predictors, potential confounders, and effect modifiers. Give diagnostic criteria, if applicable | 7-10 |
| Data sources/ measurement | 8* | For each variable of interest, give sources of data and details of methods of assessment (measurement). Describe comparability of assessment methods if there is more than one group | 7-10 |
| Bias | 9 | Describe any efforts to address potential sources of bias | na |
| Study size | 10 | Explain how the study size was arrived at | 13 |
| Quantitative variables | 11 | Explain how quantitative variables were handled in the analyses. If applicable, describe which groupings were chosen and why | na |
| Statistical methods | 12 | (*a*) Describe all statistical methods, including those used to control for confounding | 13-15 |
| | | (*b*) Describe any methods used to examine subgroups and interactions | na |
| | | (*c*) Explain how missing data were addressed | na |
| | | (*d*) If applicable, explain how loss to follow-up was addressed | na |
| | | (*e*) Describe any sensitivity analyses | na |
| **Results** | | | |

For peer review only

| | | | |
|---|---|---|---|
| Participants | 13* | (a) Report numbers of individuals at each stage of study—eg numbers potentially eligible, examined for eligibility, confirmed eligible, included in the study, completing follow-up, and analysed | na |
| | | (b) Give reasons for non-participation at each stage | na |
| | | (c) Consider use of a flow diagram | na |
| Descriptive data | 14* | (a) Give characteristics of study participants (eg demographic, clinical, social) and information on exposures and potential confounders | na |
| | | (b) Indicate number of participants with missing data for each variable of interest | na |
| | | (c) Summarise follow-up time (eg, average and total amount) | na |
| Outcome data | 15* | Report numbers of outcome events or summary measures over time | na |
| Main results | 16 | (*a*) Give unadjusted estimates and, if applicable, confounder-adjusted estimates and their precision (eg, 95% confidence interval). Make clear which confounders were adjusted for and why they were included | na |
| | | (*b*) Report category boundaries when continuous variables were categorized | na |
| | | (*c*) If relevant, consider translating estimates of relative risk into absolute risk for a meaningful time period | na |
| Other analyses | 17 | Report other analyses done—eg analyses of subgroups and interactions, and sensitivity analyses | na |
| **Discussion** | | | |
| Key results | 18 | Summarise key results with reference to study objectives | na |
| **Limitations** | | | |
| Interpretation | 20 | Give a cautious overall interpretation of results considering objectives, limitations, multiplicity of analyses, results from similar studies, and other relevant evidence | na |
| Generalisability | 21 | Discuss the generalisability (external validity) of the study results | na |
| **Other information** | | | |
| Funding | 22 | Give the source of funding and the role of the funders for the present study and, if applicable, for the original study on which the present article is based | 20 |

*Give information separately for cases and controls in case-control studies and, if applicable, for exposed and unexposed groups in cohort and cross-sectional studies.

**Note:** An Explanation and Elaboration article discusses each checklist item and gives methodological background and published examples of transparent reporting. The STROBE checklist is best used in conjunction with this article (freely available on the Web sites of PLoS Medicine at http://www.plosmedicine.org/, Annals of Internal Medicine at http://www.annals.org/, and Epidemiology at http://www.epidem.com/). Information on the STROBE Initiative is available at www.strobe-statement.org.

For peer review only
